# Small non-coding RNA profiling and the role of piRNA pathway genes in the protection of chicken primordial germ cells

**DOI:** 10.1186/1471-2164-15-757

**Published:** 2014-09-04

**Authors:** Deivendran Rengaraj, Sang In Lee, Tae Sub Park, Hong Jo Lee, Young Min Kim, Yoon Ah Sohn, Myunghee Jung, Seung-Jae Noh, Hojin Jung, Jae Yong Han

**Affiliations:** Department of Agricultural Biotechnology and Research Institute of Agriculture and Life Sciences, College of Agriculture and Life Sciences, Seoul National University, Seoul, 151-921 Korea; Department of Animal Science and Technology, Chung-Ang University, Anseong, 456-756 Korea; Graduate School of International Agricultural Technology and Institute of Green-Bio Science and Technology, Seoul National University, Pyeongchang-gun, Gangwon-do, 232-916 Korea; Codes Division, Insilicogen, Inc, Suwon, 441-813 Korea

**Keywords:** Aves, Non-coding RNA, piRNA, Primordial Germ Cells

## Abstract

**Background:**

Genes, RNAs, and proteins play important roles during germline development. However, the functions of non-coding RNAs (ncRNAs) on germline development remain unclear in avian species. Recent high-throughput techniques have identified several classes of ncRNAs, including micro RNAs (miRNAs), small-interfering RNAs (siRNAs), and PIWI-interacting RNAs (piRNAs). These ncRNAs are functionally important in the genome, however, the identification and annotation of ncRNAs in a genome is challenging. The aim of this study was to identify different types of small ncRNAs particularly piRNAs, and the role of piRNA pathway genes in the protection of chicken primordial germ cells (PGCs).

**Results:**

At first, we performed next-generation sequencing to identify ncRNAs in chicken PGCs, and we performed *ab initio* predictive analysis to identify putative piRNAs in PGCs. Then, we examined the expression of three repetitive sequence-linked piRNAs and 14 genic-transcript-linked piRNAs along with their linked genes using real-time PCR. All piRNAs and their linked genes were highly expressed in PGCs. Subsequently, we knocked down two known piRNA pathway genes of chicken, PIWI-like protein 1 (*CIWI*) and 2 (*CILI*), in PGCs using siRNAs. After knockdown of *CIWI* and *CILI*, we examined their effects on the expression of six putative piRNA-linked genes and DNA double-strand breakage in PGCs. The knockdown of *CIWI* and *CILI* upregulated chicken repetitive 1 (*CR1*) element and *RAP2B*, a member of RAS oncogene family, and increased DNA double-strand breakage in PGCs.

**Conclusions:**

Our results increase the understanding of PGC-expressed piRNAs and the role of piRNA pathway genes in the protection of germ cells.

**Electronic supplementary material:**

The online version of this article (doi:10.1186/1471-2164-15-757) contains supplementary material, which is available to authorized users.

## Background

Organisms that undergo sexual reproduction usually develop from the fusion of male and female gametes during the fertilization process. Germ cells are the only cells that produce functional gametes and transmit parental genetic information to the progeny. Primordial germ cells (PGCs) are the precursors of germ cells and are specified during the early days of embryonic development in all vertebrate species. The origin, migratory routes, and timing of PGC differentiation into germ cells vary among vertebrate species. In chickens, PGCs emerged initially in cleavage-stage embryos [1], and then migrated through the hypoblast layer, germinal crescent area, and blood vessels to reach the bilateral embryonic gonads by approximately embryonic day (E) 2.5 [1–3]. After reaching the gonads, PGCs undergo rapid mitotic cell division to increase their population number. Finally, PGCs differentiate into oogonia in females and prospermatogonia in males at approximately E8.0 and E13.0, respectively [4, 5]. Chicken PGCs can be easily isolated from the early embryos, and cultured long-term without losing their characteristic features [6]. Therefore, chicken PGCs act as an efficient tool for studying the early migration of germ cells, for identifying the germ cell-expressed gene functions, and for the production of transgenic birds [5, 7–10]. Recently, several reviews were emphasized the very recent progresses of PGC studies in biomedical sciences and animal biotechnology [11–14]. Early embryonic development, including the PGC lineage, is governed by the action of many genes and proteins. However, previous studies have shown that small non-coding RNAs (ncRNAs) do not efficiently govern PGCs in vertebrate species. ncRNAs are functional RNAs but lack coding sequences that can be translated into functional proteins. ncRNAs have been described as a broad class of regulatory RNA molecules whose functions continue to be characterized in a variety of model organisms and diseases [15]. The classification of ncRNAs includes highly abundant and functionally important transfer RNAs (tRNAs), ribosomal RNAs (rRNAs), small cytoplasmic RNA (scRNAs), small nuclear RNAs (snRNAs), small nucleolar RNAs (snoRNAs), micro RNAs (miRNAs), small-interfering RNAs (siRNAs), and P-element-induced wimpy testis (PIWI)-interacting RNAs (piRNAs) [16–18]. The sizes of most small ncRNAs range between 18 and 32 nt in length, but determining the total number of these ncRNAs in a genome remains challenging. Recently, many high-throughput technologies, including genome-wide association (GWA) studies, chromatin immunoprecipitation followed by sequencing (ChIP-seq) and RNA sequencing (RNA-seq), have been developed to examine various aspects of cellular processes, including the transcriptome, epigenome, proteome and interactome [16, 19]. Next-generation sequencing (NGS) has played an important role in genomic research and has fundamentally changed the nature of genetic experimentation [15]. NGS can be used to detect alternative splice variants using paired ends, as well as to detect relatively short reads or longer reads. In addition, NGS can detect novel classes of ncRNAs [17, 19]. In this study, we performed high-throughput NGS followed by standard annotation protocols to identify different types of small ncRNAs particularly piRNAs in chicken PGCs compared with gonadal stromal cells (GSCs) and chicken embryonic fibroblasts (CEFs), in order to verify their biofunctional activity.

## Results

### Next-generation sequencing of small ncRNAs

**Figure 1:**
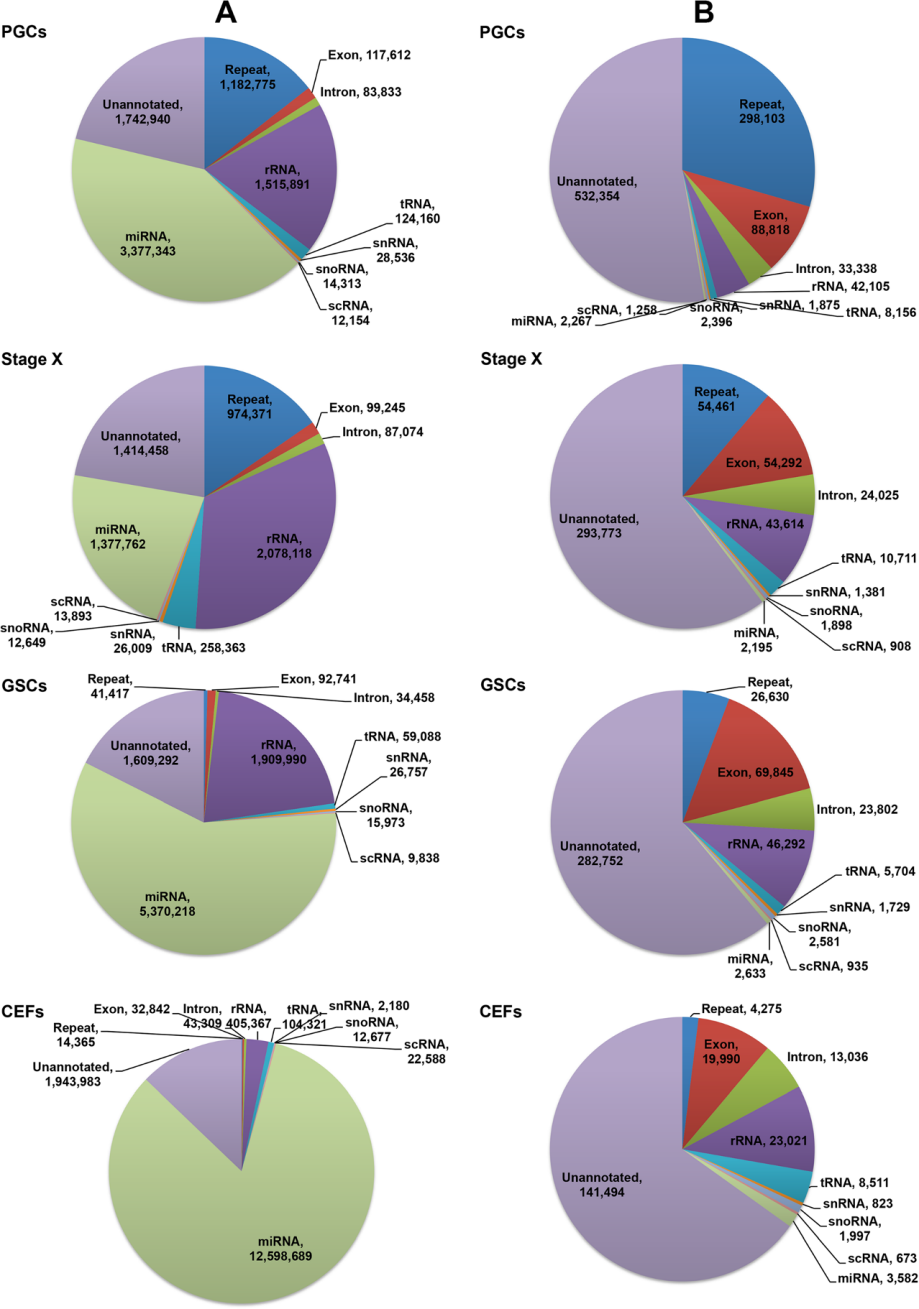
**Genome distribution of total and unique reads.** Genome distribution of total **(A)** and unique **(B)** reads in PGCs, stage X blastoderms, GSCs, and CEFs obtained using high-throughput next-generation sequencing following the standard annotation protocols.

High-throughput NGS was performed in chicken PGCs using the Illumina HiSeq platform. We maintained stage X blastoderms, GSCs, and CEFs as reference samples. Standard protocols were then followed to screen the sequencing data and annotate the small ncRNAs. Raw NGS data were processed to obtain clean reads by removing low-quality reads (Q-value < 13), short read tags (<18 nt), and adaptor-ligated contaminants. After the cleaning process, the raw data contained 8,199,557 total reads in PGCs, 6,341,942 in stage X blastoderms, 9,169,772 in GSCs, and 15,180,853 in CEFs. From these total reads, there were 1,010,670 unique reads in PGCs, 487,258 in stage X blastoderms, 462,903 in GSCs, and 217,402 in CEFs (Table 1). The statistics of sequencing quality is shown in Additional file 1: Table S1. All obtained sequence reads were matched with the current release of the chicken genome in the National Center for Biotechnology Information (NCBI, *Gallus gallus* v.4). Among the annotated reads, the most abundant total read length was ~22 nt. However, the most abundant length of the unique reads in PGCs was ~26 nt, in contrast to the other test samples (Additional file 2: Figure S1). Among the annotated reads in PGCs, the majority of unique reads were mapped to repetitive elements (298,103), followed by exon, rRNA, intron, or tRNA sequences. In stage X blastoderms, the majority of unique reads were mapped to repetitive elements (54,461), followed by exon, rRNA, intron, or tRNA sequences. In GSCs, the majority of unique reads were mapped to exons (69,845), followed by rRNA, repetitive elements, intron, or tRNA sequences. In CEFs, the majority of unique reads were mapped to rRNAs (23,021), followed by exon, intron, tRNA, or repetitive element sequences (Figure 1). The remaining unique reads were mapped to snRNAs, snoRNAs, and scRNAs, while miRNAs were found in low frequencies in all test samples (Figure 1). We compared the RPKM (reads per kilobase per million reads) values of all unique reads to identify the upregulated ncRNAs in the test samples. Based on the 2-fold cutoff value, 14,624 (55.55%) small ncRNAs originating from repeat sequences were upregulated in PGCs. In addition, 1,281 (4.87%) rRNAs were upregulated in PGCs. In stage X blastoderms, 7,395 (26.5%) upregulated sequences were small ncRNAs originating from repeat sequences, and 7,245 (25.96%) upregulated sequences were rRNAs. In GSCs, 3,141 (63.24%) upregulated sequences were rRNAs, and 324 (6.52%) upregulated sequences were miRNAs. In CEFs, 1,261 (24.59%) upregulated sequences were rRNAs, and 742 (14.47%) upregulated sequences were miRNAs. In addition, a significant number (>25%) of unannotated sequence reads were upregulated in all test samples (Table 2).

**Table 1:** **Raw and processed data of next-generation sequencing**

	Raw data		Clean data (Identity ≥ 90% and HSP coverage ≥ 95% to ***G. gallus*** v.4)				
Sample library	Number of total reads	Total base pairs	Number of total reads	Total base pairs	Minimum base pairs	Maximum base pairs	Number of unique reads
PGCs	9175177	449583673	8199557	193849279	18	44	1010670
Stage X	9243725	452942525	6341942	154246061	18	44	487258
GSCs	10314115	505391635	9169772	208574068	18	44	462903
CEFs	16705966	818592334	15180321	334940464	18	44	217402

**Table 2:** **Unique small ncRNAs that are upregulated in PGCs, stage X blastoderms, GSCs, and CEFs**

	PGCs		Stage X		GSCs		CEFs	
	(PGCs vs Stage X, GSCs, CEFs)		(Stage X vs PGCs, GSCs, CEFs)		(GSCs vs PGCs, Stage X, CEFs)		(CEFs vs PGCs, Stage X, GSCs)	
	Number of unique reads	%	Number of unique reads	%	Number of unique reads	%	Number of unique reads	%
Repeat	14624	55.55	7395	26.50	5	0.10	49	0.96
Unannotated	8394	31.88	9335	33.46	1248	25.13	2181	42.53
rRNAs	1281	4.87	7245	25.96	3141	63.24	1261	24.59
Intron sense	629	2.39	566	2.03	4	0.08	84	1.64
tRNAs	507	1.93	1831	6.56	37	0.74	554	10.80
Intron antisense	320	1.22	180	0.65	8	0.16	51	0.99
Exon sense	251	0.95	435	1.56	49	0.99	67	1.31
scRNA	92	0.35	124	0.44	20	0.40	44	0.86
miRNAs	96	0.36	448	1.61	324	6.52	742	14.47
snoRNA	75	0.28	165	0.59	96	1.93	79	1.54
snRNA	53	0.20	151	0.54	35	0.70	9	0.18
Exon antisense	6	0.02	28	0.10	0	-	7	0.14
Total	26328	100	27903	100	4967	100	5128	100

### piRNAs obtained using proTRAC software

Since the most abundant base pair size (~26 nt) and annotated classification (repeat sequence origin) of unique reads in PGCs were the potential characteristic of piRNAs, we performed *ab initio* predictive analysis using probabilistic TRacking and Analysis of Clusters (proTRAC) software to identify putative piRNAs in chicken PGCs compared with stage X blastoderms, GSCs, and CEFs. Among all annotated and unannotated unique reads, those that met the input criteria were only accepted for analysis using proTRAC software. The proTRAC output revealed 92,373 unique piRNAs in PGCs. Among the putative piRNAs, a large number (74,337) were derived from repeat sequences. The second largest number of piRNAs (14,478) was derived from unannotated sequences. Approximately 2,827 piRNAs were derived from intronic genetic sequences, and ~ 645 piRNAs from gene exons. In addition, a small proportion of other ncRNAs were identified as piRNAs. In stage X blastoderms, proTRAC output revealed 12,124 unique piRNAs, among which 10,925 were derived from repeat sequences. In GSCs, proTRAC output revealed 4,930 unique piRNAs, among which 3,116 were derived from repeat sequences. In CEFs, proTRAC output revealed only 642 unique piRNAs, and among these piRNAs the most abundant were derived from unannotated sequences (Table 3). In order to cross-validate the putative chicken piRNAs, we performed another *ab initio* predictive analysis using piRNApredictor software with the sequence reads of test samples. The piRNApredictor output revealed more number of unique piRNAs in each test sample (Additional file 3: Table S2). The output results of proTRAC and piRNApredictor were then compared. In this comparison also, a large number of piRNAs were derived from repeat sequences in PGCs. The second largest number of piRNAs was derived from unannotated sequences (Additional file 4: Table S3). However, the piRNAs identified using proTRAC were only referred in the subsequent analysis in this study.

**Table 3:** **proTRAC output and distribution of piRNAs from next-generation sequencing**

Samples	proTRAC input	proTRAC output	Distribution of piRNAs									
	Number of unique reads	Number of unique piRNAs	Repeat	Unannotated	Intron sense	Intron antisense	Exon sense	Exon antisense	snRNA	rRNA	miRNA	tRNA
PGCs	687544	92373	74337	14478	1961	866	524	121	50	22	13	1
Stage X	350113	12124	10925	925	194	63	13	1	0	2	0	1
GSCs	350647	4930	3116	1562	121	62	59	0	5	0	5	0
CEFs	159803	642	31	360	17	2	122	20	1	2	0	87

Table 4 shows the top 20 putative piRNAs originating from repeat sequences based on the RPKM values in PGCs, the majority of which were derived from the long interspersed element (*LINE*) or chicken repetitive 1 (*CR1*). Table 5 shows the top 20 putative piRNAs originating from genic sequences based on RPKM values in PGCs. We also searched for additional information on these piRNA-linked coding genes, such as their associated pathways using the Kyoto Encyclopedia of Genes and Genomes database (KEGG) [20], functional domains using protein family matrices (Pfam) [21], and molecular function gene ontologies using AmiGO, a web based application for gene ontology search [22]. Furthermore, we searched for possible functions of piRNA-linked coding genes based on earlier studies. These search results demonstrated that the majority of piRNA-linked coding genes are involved in specific pathways and are functionally related to germ cells, testis, and ovary in different vertebrates (Additional file 5: Table S4).

**Table 4:** **List of top 20 repeat sequences originated piRNAs that upregulated in PGCs**

piRNA seq. ID	Sequence length	Sequences	Origin	Genomic distribution	PGCs	Stage X	GSCs	CEFs	Fold change
					(RPKM)	(RPKM)	(RPKM)	(RPKM)	
ISG_3439104*	25	TATTTCCTAACGTCCAGCCTGAACC	Repeat seq.	*LINE/CR1:0*	1147.02	13.07	13.52	0	129.42
ISG_1952422*	27	CCAGAACACACTTGGCCTTCCGGGCTG	Repeat seq.	*LINE/CR1:0*	928.3	3.78	17.55	0	130.55
ISG_2828838	25	CACTGATGGACAGGTCCTGGCTAAG	Repeat seq.	*LTR/ERVL:1*	251.4	2.68	3.92	0	114.24
ISG_1920655*	27	ACTGAACACAGCACTCGAGGTGAGGCC	Repeat seq.	*LINE/CR1:0*	242.5	2.83	3.49	0	115.05
ISG_587310	25	TATTTCCTAACGTCCAGCCTGAATC	Repeat seq.	*Ambi*	215.07	1.73	2.29	0	160.43
ISG_434349	27	TCCTTGCACAGCCACGACAGTCGCCTG	Repeat seq.	*LINE/CR1:0*	205.56	0.31	3.6	0	157.61
ISG_3712010	25	TACCTGTAGAACCCCTTCTTGTTGT	Repeat seq.	*LINE/CR1:0*	194.22	0.63	4.03	0	124.93
ISG_347271	28	TTGAACCTCATTAGGTTTTCGTGGGACC	Repeat seq.	*LINE/CR1:0*	175.2	2.36	2.83	0	101.14
ISG_471296	27	TGCACTCGATGCCATCGTCTGTCACTG	Repeat seq.	*LINE/CR1:0*	169.35	0.47	4.03	0	112.74
ISG_2635821	26	TTCCAGCGTTGTGTGATTTTAGAAGC	Repeat seq.	*LINE/CR1:1*	155.45	0.63	3.16	0	123
ISG_2618644	26	TGCTGACGGACTTCCCTGGGCCTGCT	Repeat seq.	*LTR/ERVL:1*	150.81	0.16	1.85	0	225
ISG_3554587	29	TCTGATCATCCTCTGGACTTGCTCCAAGA	Repeat seq.	*LINE/CR1:0*	149.72	0.63	2.51	0	143.16
ISG_3050277	25	TTTTGACTTAAAAAACGTGTGCGCC	Repeat seq.	*LTR/ERVL:1*	144.48	2.99	1.09	0	106.18
ISG_1247918	25	AAGAAAGACGCAGAGCTCTTGGACC	Repeat seq.	*LINE/CR1:1*	130.09	0.79	3.05	0	101.63
ISG_2559548	26	TTTCCATCCCTCACTGTCTCTGAGCT	Repeat seq.	*LINE/CR1:0*	120.94	0.16	2.07	0	162.79
ISG_2994213	27	TGTACCTGTAGAACCCCTTCTTGTTGT	Repeat seq.	*LINE/CR1:0*	119.36	0	1.74	0	205.28
ISG_2004249	23	TTTCCTAACGTCCAGCCTGAACC	Repeat seq.	*LINE/CR1:0*	110.95	0.16	1.64	0	185.66
ISG_988698	26	GATGATCAGAGGGCTGGAGCACCTCC	Repeat seq.	*LINE/CR1:1*	110.22	0.63	1.96	0	127.55
ISG_1691836	26	TGTACCTGTAGAACCCCTTCTTGTTG	Repeat seq.	*LINE/CR1:0*	109.73	0.16	1.64	0	183.61
ISG_2499527	25	AGGAATGGGCTGCCCAGAGAGGTGG	Repeat seq.	*LINE/CR1:1*	108.39	0	1.53	0	213.04

*LINE*: long interspersed element; *CR1*: chicken repetitive 1 element; *LTR*: long terminal repeat; ERVL: endogenous retroviral element; *Ambi* (ambiguous): denotes small RNAs overlap with more than one repeat type. *piRNAs were examined by qPCR along with *CR1*.

**Table 5:** **List of top 20 genic sequences originated piRNAs that upregulated in PGCs**

piRNA seq. ID	Sequence length	Sequences	Origin	Genomic distribution	Associated gene	PGCs	Stage X	GSCs	CEFs	Fold change
						(RPKM)	(RPKM)	(RPKM)	(RPKM)	
ISG_2943457*	25	GACAGACAGAGCTGCCCCTGAGCCT	Intron_antisense	NM_001146136_intr_4	*CHIR-B5*	55.72	0	0.87	0	191.65
ISG_1259042*	27	AGACTGAAGATGTGCACCTGACGCCAG	Intron_sense	NM_001146141_intr_4	*CHIR-AB1*	49.62	0	0.76	0	195.06
ISG_2785619*	26	TTCTGCATGTTGCTCTCTGTCAGCTG	Intron_sense	NM_001030561_intr_5	*PLLP*	42.92	0	0.33	0	393.64
ISG_2237691	25	AGACTGAAGATCTGCACCTGACACC	Intron_sense	NM_001146136_intr_6	*CHIR-B5*	35.6	0	0.87	0	122.45
ISG_2022559*	27	TGTTTACTGACTGAGCTACTTTTCCCC	Intron_sense	NM_205163_intr_15	*MYO1A*	33.41	0	0.11	0	919.25
ISG_2633063*	26	AGGGTACTGAGACATCTTGGAGACAA	Intron_antisense	NM_204716_intr_4	*SLC6A2*	19.87	0	0.33	0	182.28
ISG_1354003*	21	TTTCCAAGGACCAGTAGCGCT	Intron_sense	NM_205429_intr_1	*17.5*	18.29	0	0.55	0	100.65
ISG_2087909*	26	TCTCAAAGGATTCCGCATCGTCGACG	Exon_antisense	NM_001030702_exon_1	*RAP2B*	11.95	0	0	0	1194.81
ISG_3065006*	28	AAGGACCCAAATGGTAGCAGAGGCCATG	Intron_antisense	NM_205186_intr_18	*LRP8*	9.51	0	0.11	0	261.68
ISG_3356981*	25	AATGCTGAGAACTAAGGATGCCTCC	Intron_sense	NM_205098_intr_2	*VDR*	7.92	0	0	0	792.48
ISG_3294350*	30	CAGGCTGTGACCCTGGAATTCCACTACACT	Intron_sense	NM_001013397_intr_72	*MYH1E*	7.44	0	0.11	0	204.65
ISG_2645108*	27	GAGTGTGAGAAGGGCTTTGTGCAGAGC	Exon_antisense	NM_001030695_exon_5	*ZNF302*	5.24	0	0	0	524.26
ISG_2675670*	23	CGGTGTGGGACGAGAAGGAGAAC	Exon_sense	NM_204729_exon_2	*RGN*	4.88	0	0	0	487.68
ISG_3080707*	27	CTGTGAGTGTGTGAGTGCGGCGGCGCG	Intron_antisense	NM_204952_intr_1	*FOXD2*	4.27	0	0.11	0	117.42
ISG_3280151*	24	AAGGACCTCTGAGAATTGCTTTCT	Exon_sense	NM_001030884_exon_12	*RASSF2*	2.32	0	0	0	231.65
ISG_1621199	27	CAGAAGAGAAGCTGAACACAGGGTGTC	Intron_antisense	NM_001031121_intr_7	*VAMP7*	1.95	0	0	0	195.07
ISG_882291	28	TTAAAGATATTTGGCTGCCTGGCTCGCC	Intron_sense	NM_001081502_intr_4	*NLGN1*	1.95	0	0	0	195.07
ISG_2363541	26	AAGGACACCGAGGCTCTGCGTGCTGA	Exon_sense	NM_205525_exon_2	*APOA1*	1.83	0	0	0	182.88
ISG_2033456	26	TCGGCTCGGCTCGGCTCGGCTCGGCT	Exon_antisense	NM_001006501_exon_1	*ADK*	1.1	0	0	0	109.73
ISG_950928	27	ATCTGCGTTTAAAGCTCTTTGCACACT	Intron_antisense	NM_001030561_intr_5	*PLLP*	1.1	0	0	0	109.73

*CHIR-B5*: immunoglobulin-like receptor CHIR-B5; *CHIR-AB1*: immunoglobulin-like receptor CHIR-AB1; *PLLP*: plasmolipin; *MYO1A:* myosin IA; *SLC6A2:* solute carrier family 6, member 2; *17.5*: lectin-like protein, type II transmembrane protein (17.5); *RAP2B:* RAP2B, member of RAS oncogene family; *LRP8:* low density lipoprotein receptor-related protein 8, apolipoprotein e receptor; *VDR*: vitamin D3 receptor; *MYH1E*: myosin, heavy chain 1E, skeletal muscle; *ZNF302*: zinc finger protein 302; *RGN*: regucalcin; *FOXD2*: forkhead box D2; *RASSF2*: Ras association (RalGDS/AF-6) domain family member 2; *VAMP7*: vesicle-associated membrane protein 7; *NLGN1*: neuroligin 1; *APOA1*: apolipoprotein A-I; *ADK*: adenosine kinase. *piRNAs were examined by qPCR along with their associated genes.

### Expression analysis of putative piRNAs and piRNA-linked genes

**Figure 2:**
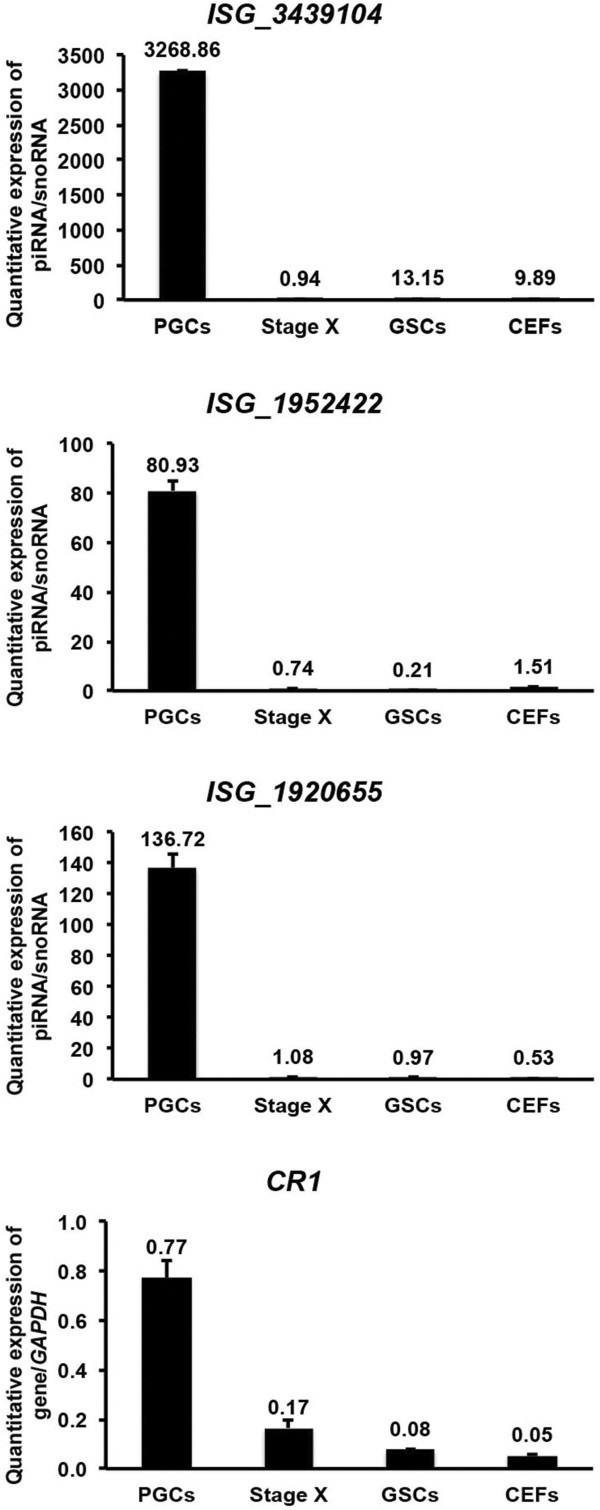
**qPCR analysis of piRNAs and piRNA-linked non-coding gene.** qPCR quantitative expression analysis of three repeat sequence-linked putative piRNAs that were upregulated in PGCs, along with *CR1*. cDNA templates used for the amplification of piRNAs and genes were prepared separately, and amplified using the appropriate piRNA- and gene-specific primers. The expression of piRNAs and *CR1* were normalized against that of snoRNA and *GAPDH*, respectively. Bars indicate the SEM of triplicate analyses.

**Figure 3:**
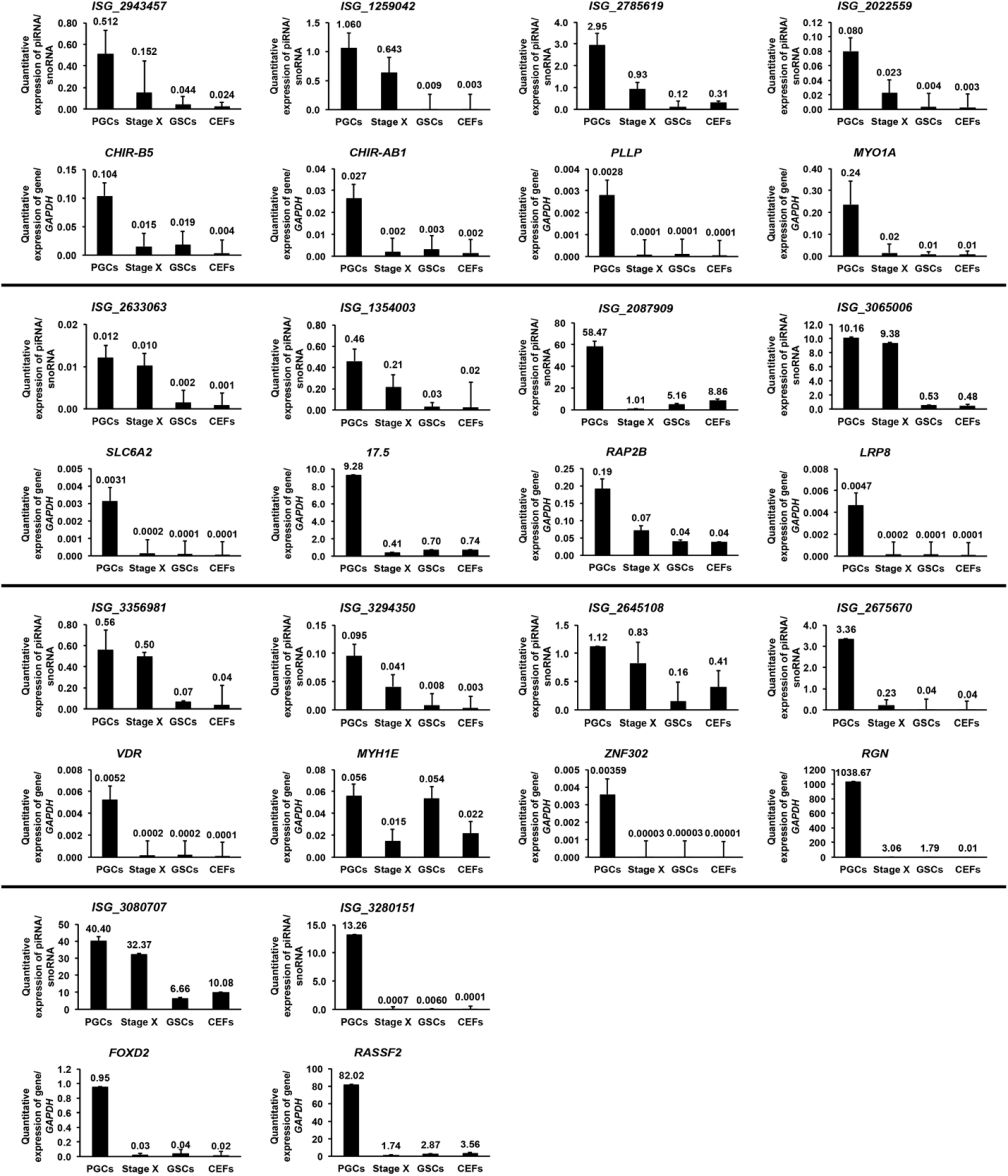
**qPCR analysis of piRNAs and piRNA-linked coding genes.** qPCR quantitative expression analysis of 14 genic-transcript-linked piRNAs that were upregulated in PGCs, along with their linked coding genes. cDNA templates used for the amplification of piRNAs and genes were prepared separately, and amplified with appropriate piRNA- and gene-specific primers. The expression of piRNAs and genes were normalized against that of snoRNA and *GAPDH*, respectively. Bars indicate the SEM of triplicate analyses.

We examined the expression of three repeat sequence-linked piRNAs (ISG_3439104, ISG_1952422, ISG_1920655) that were upregulated in PGCs (RPKM value > 2) along with *CR1* using quantitative real-time PCR (qPCR). The expression of the three repeat sequence-linked piRNAs and *CR1* were enriched in PGCs compared with stage X blastoderms, GSCs, and CEFs. However, the expression of the piRNAs was several folds higher in PGCs than that of *CR1* (Figure 2). We then performed qPCR to examine the expression of 14 genic-transcript-linked piRNAs (ISG_2943457, ISG_1259042, ISG_2785619, ISG_2022559, ISG_2633063, ISG_1354003, ISG_2087909, ISG_3065006, ISG_3356981, ISG_3294350, ISG_2645108, ISG_2675670, ISG_3080707, ISG_3280151) that were upregulated in PGCs (RPKM value > 2) along with their 14 linked genes, including immunoglobulin-like receptor CHIR-B5 (*CHIR-B5*), immunoglobulin-like receptor CHIR-AB1 (*CHIR-AB1*), plasmolipin (*PLLP*), myosin IA (*MYO1A*)*,* solute carrier family 6, member 2 (*SLC6A2*), lectin-like protein, type II transmembrane protein (*17.5*), RAP2B, member of RAS oncogene family (*RAP2B*), low density lipoprotein receptor-related protein 8, apolipoprotein e receptor (*LRP8*), vitamin D3 receptor (*VDR*), myosin, heavy chain 1E (*MYH1E*), zinc finger protein 302 (*ZNF302*), regucalcin (*RGN*), forkhead box D2 (*FOXD2*), and ras association (RalGDS/AF-6) domain family member 2 (*RASSF2*), respectively (Figure 3). As expected, all genic-transcript-linked piRNAs and their linked genes were highly expressed in PGCs compared with stage X blastoderms, GSCs, and CEFs.

### Functional validation of piRNA-linked genes

**Figure 4:**
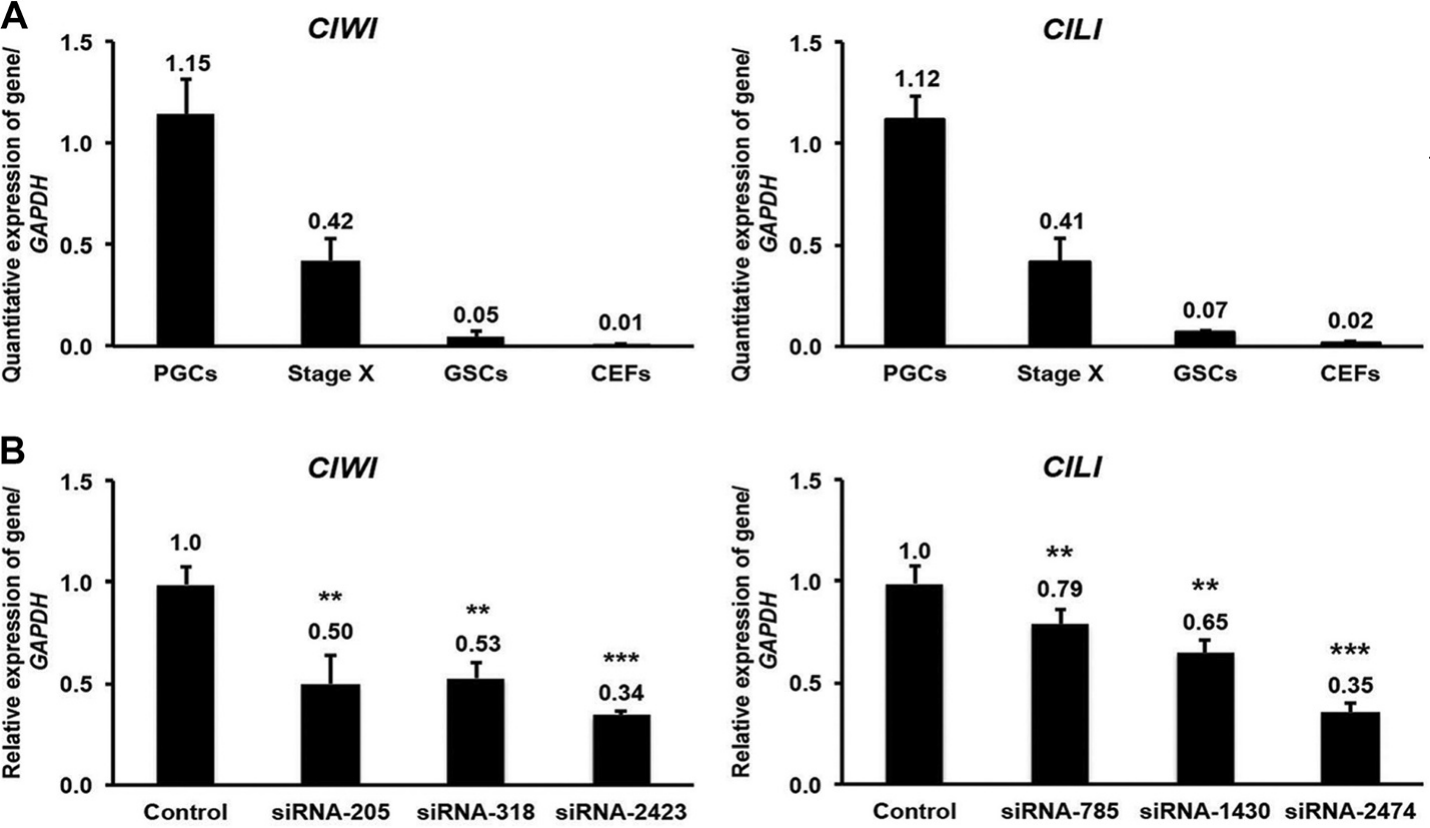
**qPCR and knockdown analysis of** ***CIWI*** **and** ***CILI*** **. (A)** qPCR quantitative expression analysis of the chicken piRNA pathway genes *CIWI* and *CILI* in PGCs, stage X blastoderms, GSCs, and CEFs. **(B)** Knockdown of *CIWI* and *CILI* in chicken PGCs. Three siRNAs each for the knockdown of *CIWI* and *CILI* were transfected into PGCs using lipofection. Approximately 48 h after transfection, knockdown efficiency was examined by qPCR amplification of *CIWI* and *CILI*. The expression of *CIWI* and *CILI* was normalized against that of *GAPDH*. Bars indicate the SEM of triplicate analyses. **P < 0.01 vs. control and ***P < 0.001 vs. control.

Since the expression of the identified piRNAs and their linked genes were higher in germ cells than in other test samples, we performed an indirect functional validation of selected piRNA-linked genes by knocking down the known testis/ovary-specific piRNA pathway genes from chicken, PIWI-like protein 1 (*CIWI*) and 2 (*CILI*). We first examined the expression patterns of *CIWI* and *CILI* using qPCR and *in situ* hybridization. The expression of *CIWI*, detected by qPCR, was several folds higher in PGCs than in stage X blastoderms, GSCs, and CEFs (Figure 4A). Furthermore, the expression of *CIWI* was detected continually in the developing germ cells at various developmental stages of male and female gonads based on *in situ* localization. In the adult stage, the expression of *CIWI* was restricted to spermatogonia in males and oocytes in females (Additional file 6: Figure S2). Similar to *CIWI*, the expression of *CILI* was also several folds higher in PGCs detected by qPCR (Figure 4A) and localized to germ cells throughout the developmental stages based on *in situ* hybridization (Additional file 7: Figure S3). However, the expression of *CILI* was slightly stronger than that of *CIWI*. In the adult stage, the expression of *CILI* was detected in spermatogonia and spermatocytes of males and in oocytes, granulosa cells and theca cells in females (Additional file 7: Figure S3).

**Figure 5:**
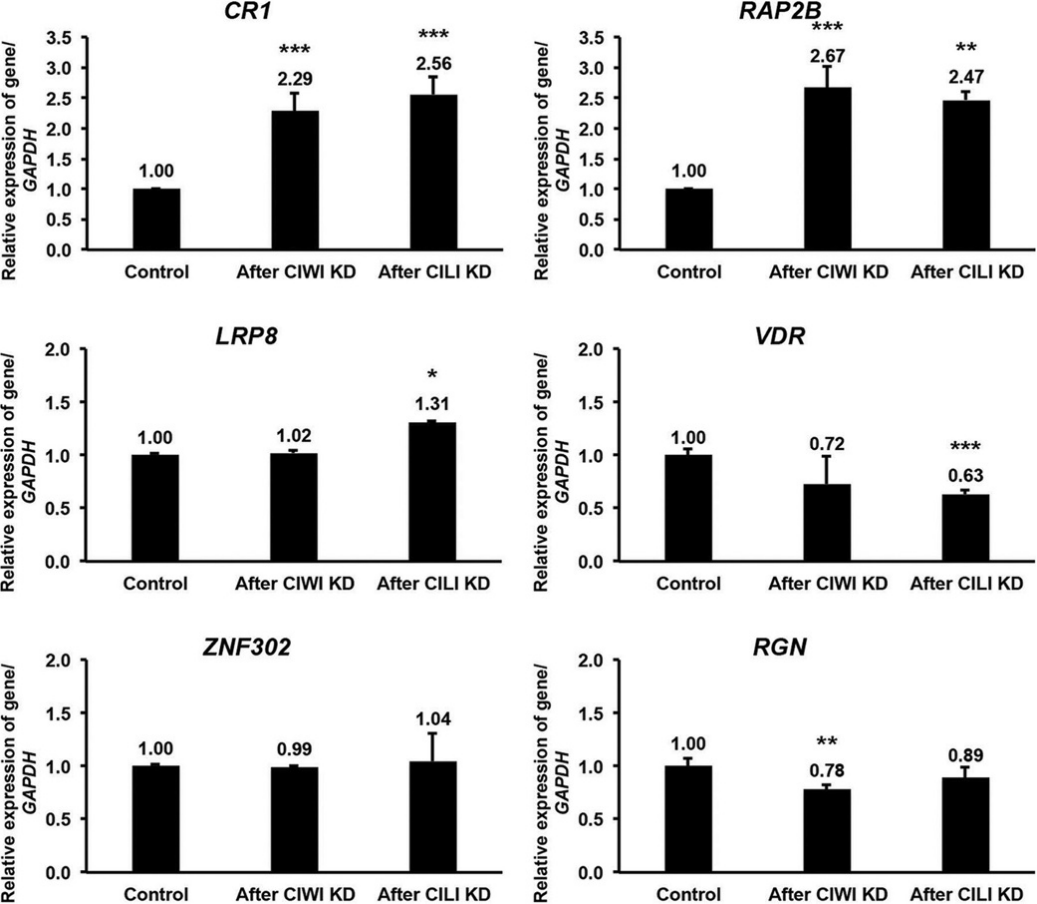
**Effects of** ***CIWI*** **and** ***CILI*** **knockdown on the expression of piRNA-linked genes.** Effects of chicken piRNA pathway genes *CIWI* and *CILI* knockdown on the expression of six putative piRNA-linked genes including *CR1*, *RAP2B*, *LRP8*, *VDR*, *ZNF302*, and *RGN* using qPCR. The relative expression of all genes was normalized against that of *GAPDH*. Bars indicate the SEM of triplicate analyses. *P < 0.05 vs. control, **P < 0.01 vs. control, and ***P < 0.001 vs. control.

We used three siRNAs (siRNA-205, siRNA-318, and siRNA-2423) to knockdown *CIWI* in PGCs using RNA transfection. Approximately 48 h after transfection, all three siRNAs significantly decreased the expression of *CIWI*: 50% decrease by siRNA-205, 47% by siRNA-318, and 66% by siRNA-2423 (Figure 4B). Similarly, we used three other siRNAs (siRNA-785, siRNA-1430, and siRNA-2474) to knockdown *CILI* and found that all three significantly decreased the expression of *CILI*: 21% decrease by siRNA-785, 35% by siRNA-1430, and 65% by siRNA-2474 (Figure 4B). After maximum knockdown of *CIWI* (using siRNA-2423) and *CILI* (using siRNA-2474) was achieved, we examined the expression of a repeat sequence originated piRNA-linked gene (*CR1*) and of five genic sequences originated piRNA-linked genes (*RAP2B*, *LRP8*, *VDR*, *ZNF302* and *RGN*) that play crucial role in germ cells. Compared with the control, knockdown of *CIWI* and *CILI* increased the expression of both *CR1* and *RAP2B* by at least 2-fold in PGCs. In contrast, knockdown of *CIWI* and *CILI* decreased the expression of *VDR* and *RGN* (Figure 5).

**Figure 6:**
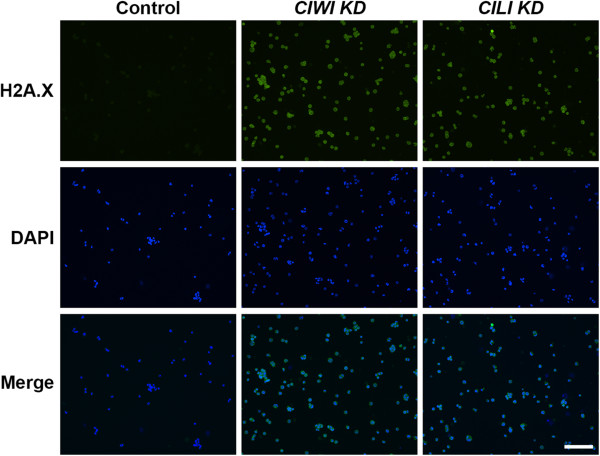
**Immunocytochemical analysis of anti-gamma H2A.X expression in PGCs after knockdown of** ***CIWI*** **and** ***CILI*** **.** Approximately 48 h after knockdown, PGCs fixed with paraformaldehyde were incubated with anti-gamma H2A.X followed by secondary antibody phycoerythrin to examine double-strand DNA breakage. DAPI was used for counterstaining. Bar = 100 μm.

Finally, we examined double-strand DNA breakage in PGCs after maximum knockdown of *CIWI* and *CILI*. Approximately 48 h after knockdown, PGCs were incubated with anti-gamma H2A.X (phospho S139) followed by incubation with phycoerythrin. Both *CIWI* and *CILI* knockdown in PGCs resulted in clear anti-gamma H2A.X staining, indicating double-strand breakage. This DNA double-strand breakage may be mediated by the increased amount of *CR1* after knockdown of *CIWI* and *CILI*. Anti-gamma H2A.X staining was not detected in control PGCs (Figure 6).

## Discussion

Protein-coding genes and proteins have been explored by several functional genomic studies in animals and plants. However, protein-coding genes account for a small proportion of the whole genome. The most exciting area in recent genomic studies has been the discovery and functional analysis of ncRNAs, which account for the majority of the genome and play critical roles in regulating protein-coding genes [23]. ncRNAs can be classified as long ncRNAs (>300 nt in length) or short ncRNAs (18-32 nt in length). Many high-throughput methods and annotation programs have been developed to identify ncRNAs. Among the high-throughput methods, the inexpensive production of large volumes of sequence data is the primary advantage of NGS over conventional methods [24]. In this study, we performed NGS to identify short ncRNAs in chicken PGCs. The data generated from high-throughput technologies can be difficult to manage [19]. Therefore, the millions of total reads obtained in this study were processed to obtain clean reads. After processing the sequences, total and unique reads were used to annotate small ncRNAs. However, a significant proportion of sequencing reads were unannotated in all test samples. Identifying ncRNA sequences and their genomic locations can be complicated, since several classes of ncRNAs are poorly conserved [15]. These unannotated reads may include novel classes of ncRNAs; therefore, their genomic conservation and potential functions on gene regulation should be explored.

In this study, annotated ncRNAs may have a specific function in an organism. For example, tRNAs and rRNAs are functionally essential for protein synthesis in all living organisms. The tRNAs of eukaryotes contain stretches of base sequences that are similar to those found in their respective rRNA, and these two RNAs share common ancestral origins rather than common functions [25]. snoRNA has diverse roles in RNA silencing, telomerase maintenance and regulation of alternative splicing. Dysregulation of snoRNAs can cause cancer in humans [26]. snRNAs transcribed from RNA polymerase II are most abundant in the nucleus of eukaryotes. snRNAs play important roles in the splicing of introns from primary genomic transcripts. Additionally, snRNAs regulate various aspects of RNA biogenesis, from transcription to polyadenylation and RNA stability [27]. miRNA is one of the best-studied classes of small RNAs. They are highly abundant and conserved among several species and involved in posttranscriptional regulation of gene expression in various tissues [16]. When we compared the RPKM values of small ncRNAs among test samples, ncRNAs originating from repeat sequences were upregulated in PGCs. In animals, siRNAs and piRNAs are mainly derived from repetitive elements (transposable elements). piRNAs are produced through the dicer-independent biogenesis pathway, which results in mature species that are longer (23-30 nt) than miRNAs and siRNAs [28]. In this report, the most abundant base pair size among the unique reads in PGCs was ~ 26 nt, a characteristic of piRNAs.

Among the classes of ncRNAs, piRNAs are important post-transcriptional regulators in germ cells. They can be classified as repetitive element sequence-derived or protein-coding genes-derived based on their genomic origins [17, 28]. Furthermore, the expression of most ncRNAs is ubiquitous, but piRNAs are expressed specifically in germ cells [17]. However, little information is available on piRNAs and their roles in germ cells of vertebrate species. In this report, we examined putative piRNAs in PGCs compared with stage X blastoderms, GSCs and CEFs. The proTRAC output showed a higher proportion of piRNAs (74,337) that were exclusively derived from different types of repeat sequences in PGCs. In addition, a significant proportion of piRNAs were derived from genomic regions and other types of ncRNAs in PGCs. Since the proTRAC software identifies piRNAs based on typical characteristics including the number of loci with T at position 1 or A at position 10 that is the so-called ping-pong signature, all identified piRNAs in chicken PGCs might be amplified in the ping-pong cycle. When we examined the expression of *CR1* and three repeat sequence-linked piRNAs, piRNA expression was high in PGCs compared with *CR1* expression. This result indicates that these piRNAs are already activated to control *CR1* expression. Based on the expression of 14 genic-transcript-linked piRNAs along with their linked genes, the expression of most piRNAs and piRNA-linked genes were highly expressed in PGCs. This may indicate that these piRNAs are produced from their linked genes to regulate various PGC functions. Nevertheless, further investigation of the co-expression of piRNAs and their linked genes in PGCs is required. In this report, we examined pathway information, functional domains and functions of piRNA-linked genes. We found that the majority of piRNA-linked genes are functionally related to germ cells, testis, and ovary in different vertebrates. piRNAs are thought to be required for germ cell development in vertebrate species [16]. Not only piRNAs but also piRNA-linked genes showed predominant functions in the germ cells of vertebrate species [29, 30]. In addition, the expression level of specific piRNAs in stage X blastoderms was very similar to those in PGCs. These piRNAs may be localized to PGCs within stage X blastoderms, which is indicative of a post-zygotic origin [28]. Numerous genes in piRNA functional pathways have been identified, including *PIWI*/Argonaute family genes, tudor family genes, maelstrom homolog, and vasa homolog [31]. piRNA pathway genes play crucial roles in germline determination during meiosis, gametogenesis, and transposon silencing. These functions may involve piRNAs and may be achieved via RNA interference silencing complex (RISC) mediated epigenetic and posttranscriptional regulation [29, 32]. In *Drosophila*, PIWI, aubergine, or spindle-E regulates epigenetic function via heterochromatin proteins (HP1 and HP2), which participates in the formation of heterochromatin along with histone H3 lysine 9 methyltransferase [29, 33]. Silencing these genes in *Drosophila* caused loss of heterochromatin formation by reduction of histone methyltransferase, and delocalization of HP1 and HP2 [33]. Similarly, fission yeast lacking an RISC component argonaute, dicer, or RNA-dependent RNA polymerase caused loss of histone methyltransferase and HP1 homolog at the centromeric heterochromatin [32, 34].

We selected chicken homologs of two piRNA pathway genes, *CIWI* and *CILI*, for indirect functional validation of the putative piRNA-linked genes obtained in this study. To our knowledge, these two PIWI family members are only identified in chicken genome, and their expression pattern in PGCs was described in our recent study [30]. We used gene-specific siRNAs to knockdown *CIWI* and *CILI*. After knockdown, we examined the expression of *CR1* and five piRNA-linked coding genes (*RAP2B*, *LRP8*, *VDR*, *ZNF302* and *RGN*). These genes were selected based on their crucial roles in germ cells. The maximal knockdown of *CIWI* and *CILI* significantly increased the expression of *CR1* and *RAP2B* in PGCs. piRNAs and their linked genes play a major defense role against transposable elements. The over activation of transposable elements is associated with severely impaired gametogenesis and causes DNA double-strand breakage in germ cells [35]. According to previous studies, increased *CR1* expression causes DNA damage in PGCs. Therefore, the expression of *RAP2B* increased under these conditions. *RAP2B* is a member of the Ras superfamily that protects cells from DNA damage in a p53-dependent manner [36]. In contrast, knockdown of *CIWI* and *CILI* decreased the expression of *VDR* and *RGN* in PGCs, which could impair germ cell development. *VDR* is crucial for vitamin D3 metabolism, Ca^2+^ homeostasis, and gametogenesis [37–40], and *RGN* is crucial for Ca^2+^ homeostasis and gametogenesis [41, 42]. The nucleosome is composed of DNA and multiple histone protein families, H2A, H2B, H3, and H4. In mammals, the H2A family is consists of three members, H2A.1-H2A.2, H2A.Z, and H2A.X. Among these three members of H2A family, H2A.X becomes phosphorylated on residue serine 139 (the site of gamma-phosphorylation) in cells when double-stranded breaks are introduced into the DNA [43, 44]. Thus, accumulation of gamma H2A.X near break site is a rapid cellular response to the presence of DNA double-strand breakage [43]. It has been reported that the aubergine, spindle-E, and armitage mutations lead to germline-specific accumulation of gamma-H2A.X in *Drosophila*[45]. In addition, gamma H2A.X accumulates in normal chromosomes where the meiotic double-strand breaks are formed [46]. In this study, to further confirm the DNA double-strand breakage in PGCs, cells were subjected to H2A.X staining after knockdown of *CIWI* and *CILI*. Our results clearly indicated that PGCs undergo *CR1*-mediated DNA double-strand breakage after knockdown of *CIWI* and *CILI*. These results suggested that *CIWI* and *CILI* interact with certain piRNA-linked genes to protect germ cells.

## Conclusions

In this study, we identified different types of ncRNAs including piRNAs in chicken PGCs. piRNAs are closely related with piRNA pathway genes, and functions specifically in germ cells. After knockdown of piRNA pathway genes *CIWI* and *CILI*, we found a transposable element-mediated DNA double-strand breakage in chicken PGCs. Several piRNA-linked genes that play crucial role in germ cells were also altered in chicken PGCs. Thus, our results significantly indicate the role of piRNA pathway genes in the protection of germ cells.

## Methods

### Experimental animals and animal care

The care and experimental use of White Leghorn (WL) chickens was approved (SNU-070823-5) by the Institute of Laboratory Animal Resources, Seoul National University, Korea. For fertilized eggs, chickens were maintained according to a standard management program at the University Animal Farm. The procedures for animal management, reproduction, and embryo manipulation adhered to the standard operating protocols of our laboratory.

### Sample preparation

Blastodermal cells at Eyal*-*Giladi and Kochav (EG&K) stage X [47] were collected by gentle dissociation of stage X blastoderms (n = 30) from freshly laid eggs in phosphate buffered saline (PBS) followed by centrifugation at 1,250 rpm for 10 min. Fertilized eggs were incubated at 37.5°C under 50–60% relative humidity. PGCs and GSCs were isolated from the gonads of chicken embryos at E6.0 (mixed sex, n = ~2,000) using the magnetic-activated cell-sorter (MACS) method [48]. Gonads were dissociated by gentle pipetting in 0.25% trypsin (Invitrogen) and 0.05% ethylenediaminetetraacetic acid (EDTA). After adding 10% fetal bovine serum (FBS, Thermo Fisher Scientific) to inactivate the trypsin-EDTA and briefly centrifuging at 200 × *g* for 5 min, total gonadal cells were incubated with anti-stage specific embryonic antigen (SSEA-1, Santa Cruz Biotechnology) for 20 min at room temperature. Cells were washed with 1 mL MACS buffer (0.5% BSA and 2 mM EDTA in PBS), and the supernatant was completely removed by centrifugation. The pellet was mixed with 100 μL MACS buffer supplemented with 20 μL goat anti-mouse IgM microbeads for 15 min at 4°C. Cells were washed with 500 μL MACS buffer and loaded onto a MACS column (Miltenyi Biotec GmbH). After several batches of cell preparation, the PGC-enriched fraction and GSCs were separated. The survival rate of PGCs purified by MACS is shown in Additional file 8: Table S5. CEFs were collected by dissociating the embryonic body (E6.0, n = 6) in 0.25% EDTA at 37°C for 20 min. Cells were then cultured in Dulbecco’s modified Eagle’s medium (DMEM; Thermo Fisher Scientific) containing 10% FBS and 1% antibiotic–antimycotic (Invitrogen) in a 5% CO_2_ atmosphere at 37°C. In addition, we collected the left gonads at E13.5, E15.5, and E17.5 and the testis and ovary at 1 day and 24 weeks from male and female chicken embryos or chickens (n = 3), respectively. All experiments discussed hereafter were performed at least in triplicate.

### High-throughput next-generation sequencing

Approximately 5 μg total RNA from each test sample (PGCs, stage X blastoderms, GSCs, and CEFs) was used to generate high-throughput NGS data using the Illumina HiSeq (Beijing Genomics Institute, China) following standard protocols from Insilicogen, Korea. Raw NGS data were processed to obtain clean reads by removing low-quality reads (Q-value < 13), short read tags (<18 nt), and adaptor-ligated contaminants using TRIM software developed by BGI for the analysis of high-throughput sequencing. To further identify sequence reads with reliable chromosomal locations, they were mapped onto the reference genome from the NCBI (*Gallus gallus* v.4) using BLAST, with identity ≥ 90% and HSP coverage ≥ 95%. Among the clean reads, those with sequence lengths between 18 and 44 nt were selected as potential small ncRNAs. Basic annotations of small ncRNAs were performed following standard protocols from Insilicogen. Briefly, to identify miRNAs, sequence reads were aligned with the precursor or mature miRNAs of chickens in miRBase19 using BLASTN with an e-value cutoff of 0.01. Sequence reads from repeat regions were identified by alignment with the reference chicken repeat sequences. To identify rRNAs, tRNAs, snoRNAs, scRNAs and snRNAs, sequence reads were aligned with the reference nucleotides from GenBank records and into the Rfam database using BLASTN with an e-value of 0.01. Sequence reads from exonic or intronic regions were identified based on their genomic locations in 17,767 reference gene sets. Furthermore, a priority rule was applied to ensure that every unique small ncRNA was mapped to only one annotation: tRNAs, rRNAs, snRNAs, snoRNAs, scRNAs > miRNAs > repeats > exons > introns.

### Prediction of piRNAs using proTRAC software

To obtain the putative chicken piRNAs, we performed *ab initio* predictive analysis using proTRAC software [49] with the sequence reads of test samples. proTRAC predicts potential piRNAs based on typical characteristics such as strand bias, the number of loci with T at position 1 or A at position 10 that is the so-called ping-pong signature, the number of loci within the typical piRNA length (26-32 nt), and the quantity of loci from infrequently mapped reads. The alignment output of sequence reads against the chicken reference genome was generated using SeqMap software [50] with the recommended ELAND3 output option, which was used as the input for proTRAC with default parameters (excluding minimum loci per cluster as 6, significance level of p ≤ 0.05 for increased hit density, and a minimum score of 1.3 for strand bias). To cross-validate the putative chicken piRNAs, we performed another *ab initio* predictive analysis using piRNApredictor software [51] with the sequence reads of test samples. The output results of proTRAC and piRNApredictor were then compared to control the false positive rate.

### qPCR analysis of piRNAs and mRNAs

qPCR was performed to examine the expression of 3 repeat sequence-linked putative piRNAs and 14 genic-transcript-linked putative piRNAs along with their 15 linked genes in PGCs, stage X blastoderms, GSCs, and CEFs. Total RNA of the test samples was isolated using TRIzol reagent (Invitrogen). For piRNA amplification, 1 μg total RNA was reverse transcribed using a miRNA 1^st^-strand cDNA synthesis kit (Agilent Technologies). To elongate the piRNAs, total RNA was first treated with *Escherichia coli* poly-A polymerase to generate a poly-A tail at the 3′-end of each RNA molecule. Following polyadenylation, cDNAs were synthesized using the RT adaptor primer. PCR was performed using the High-Specificity miRNA qPCR Core Reagent Kit (Agilent Technologies). The PCR reaction mixture was prepared by adding 2.5 μL of 10× core PCR buffer, 2.75 μL of 50 mM MgCl_2_, 10 μL of 20 mM dNTPs, 1.25 μL of 20× Eva green (Biotium), 1.0 μL of 3.125 μM piRNA-specific forward primers, 1.0 μL of 3.125 μM universal reverse primer (Agilent Technologies), 0.5 μL of High-Specificity polymerase, and 2.0 μL of cDNA to a final volume of 25 μL. PCR was performed with an initial incubation at 94°C for 10 min, followed by 40 cycles at 94°C for 10 s, 60°C for 15 s, and 72°C for 20 s. The forward primer for each piRNA, and chicken snoRNA (U24, GenBank: Z48762) was designed according to the guidelines of Agilent Technologies (Additional file 9: Table S6). piRNA expression was normalized to that of chicken snoRNA, which already validated as internal control [52].

For mRNA amplification, 1 μg total RNA was reverse transcribed using the Superscript III First-Strand Synthesis System (Invitrogen). The PCR reaction mixture was prepared by adding 2 μL PCR buffer, 1.6 μL 2.5 mM dNTP, 10 pmol each forward and reverse primer, 1 μL 20× Eva green, 0.2 μL Taq DNA polymerase, and 2 μL cDNA to a final volume of 20 μL. PCR was performed with an initial incubation at 94°C for 3 min, followed by 40 cycles at 94°C for 30 s, 60°C for 30 s, and 72°C for 30 s. qPCR primers for each target gene, and chicken glyceraldehyde-3-phosphate dehydrogenase (*GAPDH*) were designed using the Primer3 program (Additional file 10: Table S7). mRNA expression was normalized to that of chicken *GAPDH*. qPCR analysis for piRNAs and mRNAs was performed using the CFX96 real-time PCR detection system with a C1000 thermal cycler (Bio-Rad Laboratories).

### Expression analysis of *CIWI*and *CILI*

The expression patterns of two known piRNA pathway genes from chicken, *CIWI* and *CILI*, were examined using qPCR and *in situ* hybridization. For qPCR, cDNA samples from PGCs, stage X blastoderms, GSCs, and CEFs were amplified using the appropriate *CIWI* and *CILI* primers (Additional file 10: Table S7), as described above. For *in situ* hybridization, cDNA from PGCs was amplified using primers targeting *CIWI* (F: 5′-CCT GAT GGT GTA GGA GAT GGA; R: 5′-CAA GGA AAG CCA GTT TAT GGG) [GenBank: NM_001098852] and *CILI* (F: 5′-TGA GCC CCG ACA TCC ACA G; R: 5′-TTC TTG GGC AGG CAG TGG TT) [GenBank: JN248386]. The *CIWI* and *CILI* PCR products were cloned into the pGEM-T plasmid vector (Promega) and transformed into *E. coli* strain DH5α. After we verified the cloned sequence, the recombinant plasmid containing *CIWI* and *CILI* was amplified using T7- and SP6-specific primers and was subjected to cRNA probe preparation using a digoxigenin RNA labeling kit (Roche Diagnostics). Localization of *CIWI* and *CILI* during limited stages of germ cell development (E13.5, E15.5, E17.5, 1 day, and 24 weeks) in male and female chickens was examined as described previously [53]. The mRNA signal was visualized as a brown color, and images were captured under a Zeiss Axiophot light microscope (Carl Zeiss).

### Knockdown of *CIWI*and *CILI*and their effects on putative piRNA-linked genes

Gonadal PGCs were cultured briefly for mass production as described previously [8]. siRNAs targeting *CIWI* and *CILI* were designed using the RNAi designer tool (Invitrogen), which includes a sequence similarity search of the input sequence using the BLAST program. Three siRNA sequences, siRNA-205 (sense: 5′-AGA CAC UAG GAU UAC AGA U; antisense: 5′-AUC UGU AAU CCU AGU GUC U), siRNA-318 (sense: 5′-CAC GUU AGA GAA UCA AAA A; antisense: 5′-UUU UUG AUU CUC UAA CGU G), and siRNA-2423 (sense: 5′-ACU GAA ACC AGA UCA UGU A; antisense: 5′-UAC AUG AUC UGG UUU CAG U), against *CIWI* were synthesized with incorporation of a 5′-fluorescein isothiocynate (FITC) modification. In addition, three siRNA sequences, siRNA-785 (sense: 5′-GAA UUU GGU GGC UCU GCU G; antisense: 5′-CAG CAG AGC CAC CAA AUU C), siRNA-1430 (sense: 5′- AUG CUU CGA CAC CUU CGA G; antisense: 5′-CUC GAA GGU GUC GAA GCA U), and siRNA-2474 (sense: 5′-AUG CUU CGA CAC CUU CGA G; antisense: 5′-CUC GAA GGU GUC GAA GCA U), against *CILI* were synthesized. Commercially available control siRNA (sense: 5′-CCU ACG CCA CCA AUU UCG U; antisense: 5′-GGA UGC GGU GGU UAA AGC A), which was not a scrambled sequence of the test siRNAs, was purchased from Bioneer Corporation (Daejeon, Korea). To knockdown the genes, siRNAs were transfected into PGCs (500 pmol siRNA in lipofectamine + Opti-MEM per 1 × 10^5^ cells) as described previously [52]. After transfection for 48 h, FITC expression was confirmed in PGCs, and total RNA was extracted using TRIzol reagent. The efficiency of *CIWI* and *CILI* knockdown, and their effects on the expression of six candidate putative piRNA-linked genes including *CR1*, *RAP2B*, *LRP8*, *VDR*, *ZNF302*, and *RGN* were measured using qPCR.

### Immunocytochemical analysis

Immunocytochemistry was performed to examine DNA double-strand breakage in *CIWI* and *CILI* knockdown PGCs. Approximately 48 h after knockdown, PGCs were fixed in 3.7% paraformaldehyde and incubated with 1:200 diluted rabbit polyclonal to gamma H2A.X (phospho S139) antibodies (Abcam, ab11174) overnight at 4°C. After washing with PBS, PGCs were incubated with secondary antibody labeled with phycoerythrin (anti-rabbit IgG, Santa Cruz Biotechnology) for 1 h at room temperature. Cells were finally mounted with Vectashield mounting medium with 4′,6-diamidino-2-phenylindole (DAPI, Vector Laboratories), and analyzed under a fluorescence microscope (Nikon Corporation).

### Statistical analysis

Statistical analysis in gene knockdown experiments was performed using the Student’s *t* test of the SAS software (SAS Institute). Significant differences between control and treatments were analyzed using the general linear model (PROC-GLM) of the SAS software. Statistical significance was ranked as *P < 0.05, **P < 0.01, or ***P < 0.001.

### Supporting data

The raw and processed data of this project have been deposited to the GEO database (http://www.ncbi.nlm.nih.gov/geo/) under the accession number GSE60400.

## Electronic supplementary material

Additional file 1: Table S1: The statistics of sequencing quality. (PDF 41 KB)

Additional file 2: Figure S1: Size distribution of total and unique reads. Size distribution of total **(A)** and unique **(B)** reads in PGCs, stage X blastoderms, GSCs, and CEFs obtained using high-throughput next-generation sequencing following the standard annotation protocols. (TIFF 2 MB)

Additional file 3: Table S2: piRNApredictor output and distribution of piRNAs from next-generation sequencing. (PDF 42 KB)

Additional file 4: Table S3: Comparison of proTRAC and piRNApredictor outputs. (PDF 42 KB)

Additional file 5: Table S4: Pathways and functional information of genic sequences originated piRNA-associated genes. (PDF 149 KB)

Additional file 6: Figure S2: mRNA localization of *CIWI*. mRNA localization of chicken piRNA pathway gene *CIWI* during limited stages of germ cell development in male and female chickens. S: sense control. Bar = 200 μm (1^st^ and 3^rd^ columns) and 50 μm (2^nd^ and 4^th^ columns). (TIFF 8 MB)

Additional file 7: Figure S3: mRNA localization of *CILI*. mRNA localization of chicken piRNA pathway gene *CILI* during limited stages of germ cell development in male and female chickens. S: sense control. Bar = 200 μm (1^st^ and 3^rd^ columns) and 50 μm (2^nd^ and 4^th^ columns). (TIFF 9 MB)

Additional file 8: Table S5: Survival rate of PGCs purified by MACS. (PDF 39 KB)

Additional file 9: Table S6: qPCR primers used for the amplification of piRNAs. (PDF 41 KB)

Additional file 10: Table S7: qPCR primers used for the amplification of genes. (PDF 78 KB)
