# Supplementary material for: Small non-coding RNA profiling and the role of piRNA pathway genes in the protection of chicken primordial germ cells
Source: BMC Genomics. 2014 Sep 4;15(1):757. doi: 10.1186/1471-2164-15-757 (PMC4286946; doi:10.1186/1471-2164-15-757)
Supplement: Supplementary file 1 — Additional file 1: Table S1: The statistics of sequencing quality. (PDF 41 KB) [file 12864_2014_6778_MOESM1_ESM.pdf]

Table S1. The statistics of sequencing quality.

	Raw data		Sequencing quality		
Sample	Number of total reads	Total base pairs (%)	>Q10 base pairs (%)	>Q20 base pairs (%)	>Q30 base pairs (%)
PGCs	9175177	449583673 (100%)	446499729 (99.3%)	443029508 (98.5%)	437364277 (97.3%)
Stage X	9243725	452942525 (100%)	449308999 (99.2%)	445330516 (98.3%)	437482641 (96.6%)
GSCs	10314115	505391635 (100%)	501960419 (99.3%)	497913287 (98.5%)	491262999 (97.2%)
CEFs	16705966	818592334 (100%)	814478919 (99.5%)	809925849 (98.9%)	792138467 (96.8%)
