# Supplementary figures and images for: Small non-coding RNA profiling and the role of piRNA pathway genes in the protection of chicken primordial germ cells
Source: BMC Genomics. 2014 Sep 4;15(1):757. doi: 10.1186/1471-2164-15-757 (PMC4286946; doi:10.1186/1471-2164-15-757)

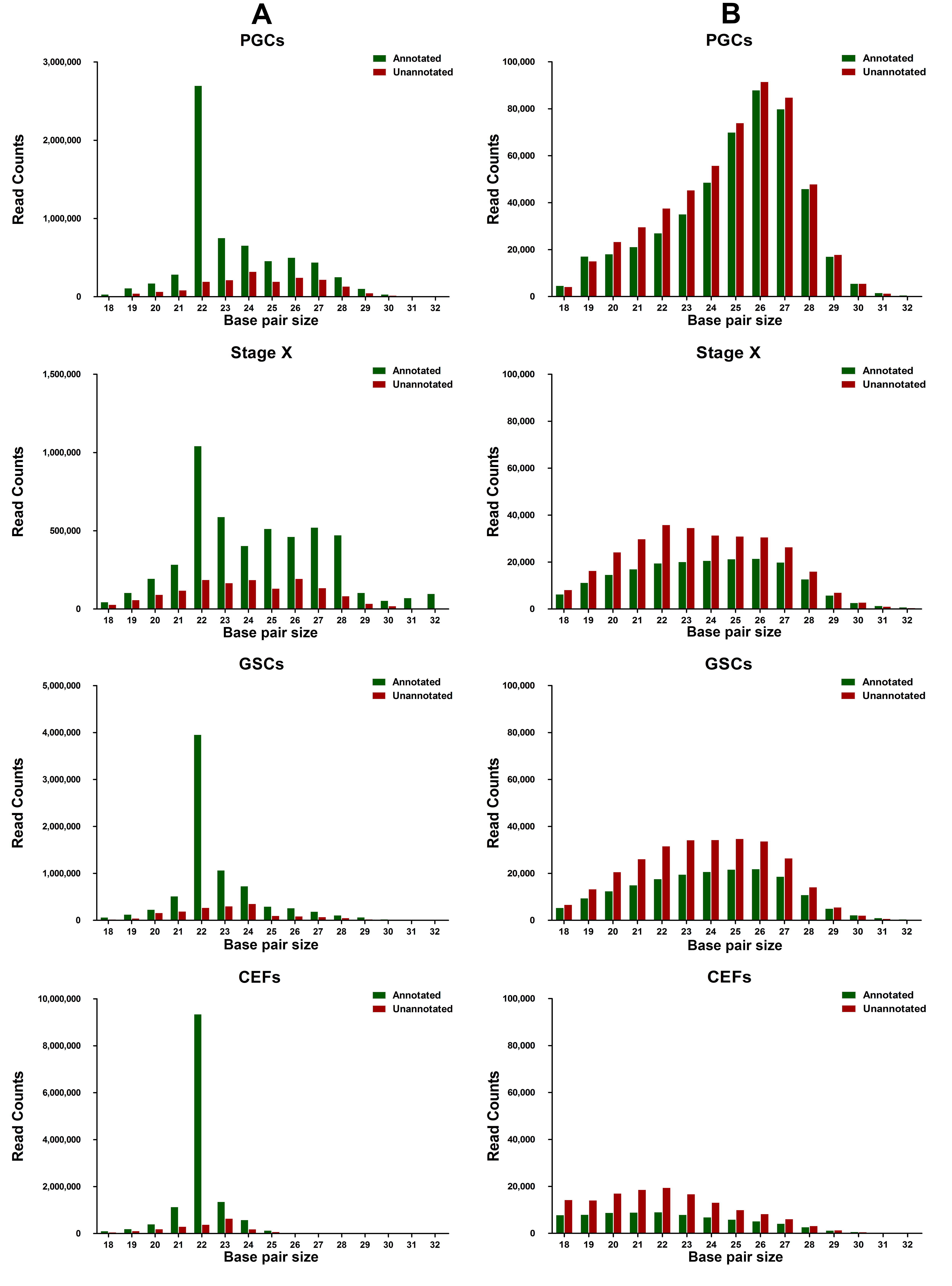

Supplement: Supplementary file 2 — Additional file 2: Figure S1: Size distribution of total and unique reads. Size distribution of total (A) and unique (B) reads in PGCs, stage X blastoderms, GSCs, and CEFs obtained using high-throughput next-generation sequencing following the standard annotation protocols. (TIFF 2 MB) [file 12864_2014_6778_MOESM2_ESM.tiff]

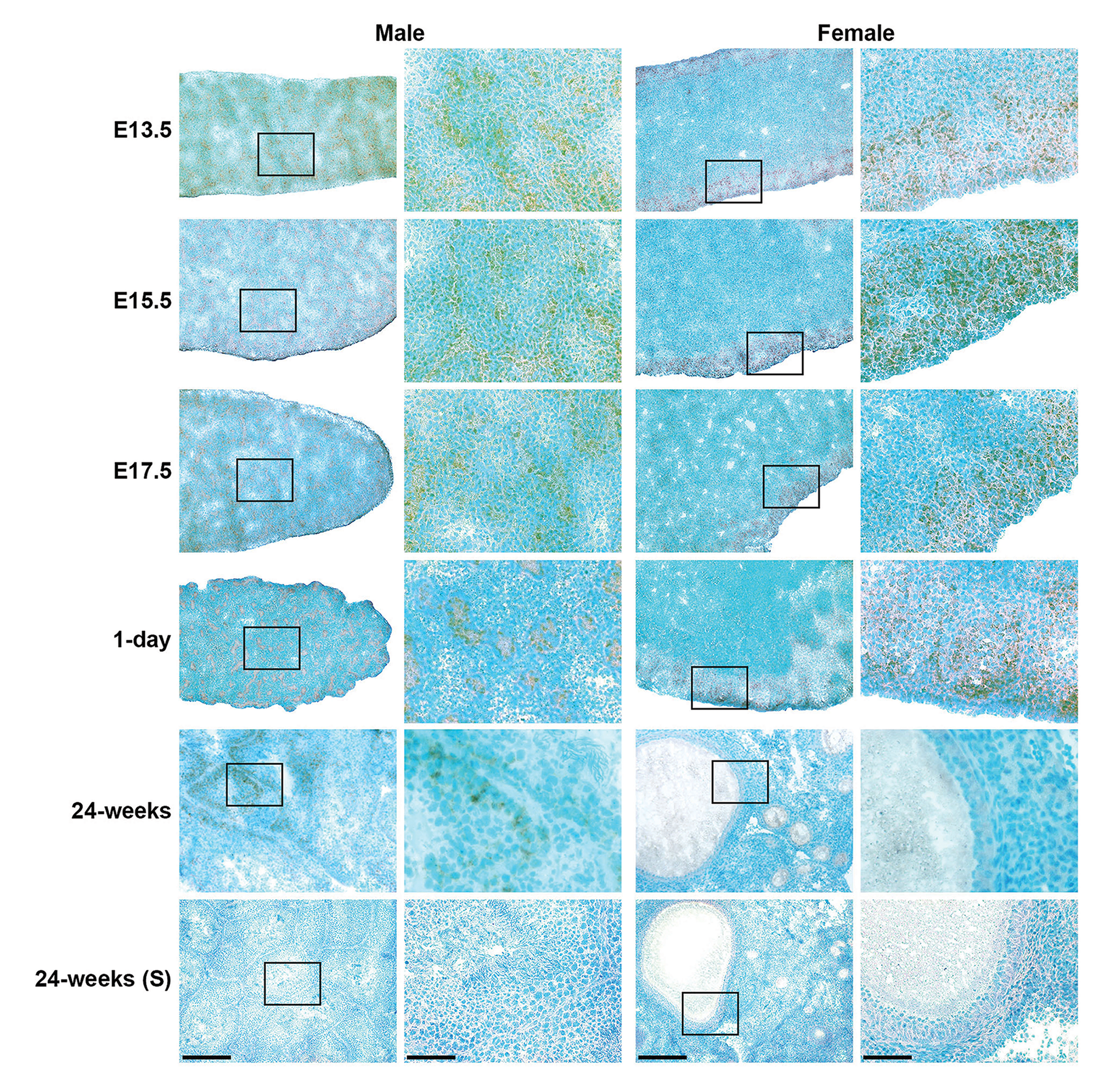

Supplement: Supplementary file 6 — Additional file 6: Figure S2: mRNA localization of CIWI. mRNA localization of chicken piRNA pathway gene CIWI during limited stages of germ cell development in male and female chickens. S: sense control. Bar = 200 μm (1st and 3rd columns) and 50 μm (2nd and 4th columns). (TIFF 8 MB) [file 12864_2014_6778_MOESM6_ESM.tiff]

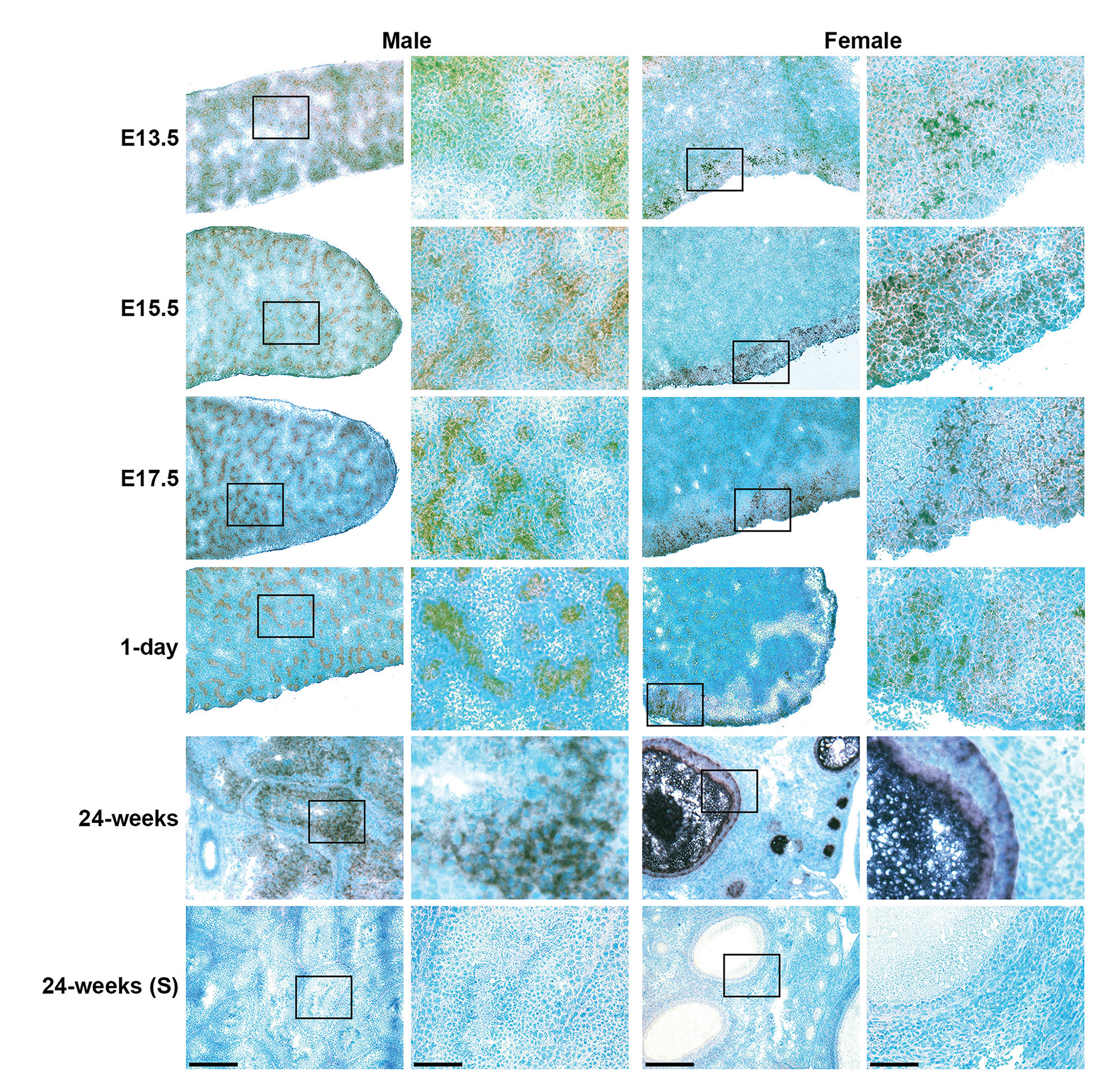

Supplement: Supplementary file 7 — Additional file 7: Figure S3: mRNA localization of CILI. mRNA localization of chicken piRNA pathway gene CILI during limited stages of germ cell development in male and female chickens. S: sense control. Bar = 200 μm (1st and 3rd columns) and 50 μm (2nd and 4th columns). (TIFF 9 MB) [file 12864_2014_6778_MOESM7_ESM.tiff]
