# Supplementary material for: Small non-coding RNA profiling and the role of piRNA pathway genes in the protection of chicken primordial germ cells
Source: BMC Genomics. 2014 Sep 4;15(1):757. doi: 10.1186/1471-2164-15-757 (PMC4286946; doi:10.1186/1471-2164-15-757)
Supplement: Supplementary file 3 — Additional file 3: Table S2: piRNApredictor output and distribution of piRNAs from next-generation sequencing. (PDF 42 KB) [file 12864_2014_6778_MOESM3_ESM.pdf]

Table S2. piRNApredictor output and distribution of piRNAs from next-generation sequencing.

| Samples | Input | Output | Distribution of piRNAs | | | | | | | | | |
|---|---|---|---|---|---|---|---|---|---|---|---|---|
| | Number of unique reads | Number of unique piRNAs | Repeat | Unannotated | Intron sense | Intron antisense | Exon sense | Exon antisense | snRNA | rRNA | miRNA | tRNA |
| PGCs | 687544 | 197237 | 104898 | 68096 | 4380 | 1542 | 15218 | 329 | 170 | 1599 | 64 | 397 |
| Stage X | 350113 | 53890 | 14820 | 25529 | 3126 | 366 | 7297 | 83 | 78 | 1629 | 51 | 504 |
| GSCs | 350647 | 57311 | 7742 | 32008 | 3355 | 331 | 11133 | 62 | 114 | 1631 | 64 | 301 |
| CEFs | 159803 | 9931 | 293 | 5816 | 766 | 133 | 1441 | 16 | 26 | 777 | 78 | 289 |
