# Supplementary material for: Small non-coding RNA profiling and the role of piRNA pathway genes in the protection of chicken primordial germ cells
Source: BMC Genomics. 2014 Sep 4;15(1):757. doi: 10.1186/1471-2164-15-757 (PMC4286946; doi:10.1186/1471-2164-15-757)
Supplement: Supplementary file 4 — Additional file 4: Table S3: Comparison of proTRAC and piRNApredictor outputs. (PDF 42 KB) [file 12864_2014_6778_MOESM4_ESM.pdf]

Table S3. Comparison of proTRAC and piRNApredictor outputs.

Samples	Input	Common Output	Distribution of common piRNAs									
	Number of unique reads	Number of unique piRNAs	Repeat	Unannotated	Intron sense	Intron antisense	Exon sense	Exon antisense	snRNA	rRNA	miRNA	tRNA
PGCs	687544	35538	28520	5845	633	259	213	46	20	1	1	0
Stage X	350113	4097	3695	301	65	31	4	0	0	1	0	0
GSCs	350647	1795	1162	562	38	23	8	0	1	0	1	0
CEFs	159803	27	1	19	3	0	4	0	0	0	0	0
