# Supplementary material for: Small non-coding RNA profiling and the role of piRNA pathway genes in the protection of chicken primordial germ cells
Source: BMC Genomics. 2014 Sep 4;15(1):757. doi: 10.1186/1471-2164-15-757 (PMC4286946; doi:10.1186/1471-2164-15-757)
Supplement: Supplementary file 5 — Additional file 5: Table S4: Pathways and functional information of genic sequences originated piRNA-associated genes. (PDF 149 KB) [file 12864_2014_6778_MOESM5_ESM.pdf]

Table S4. Pathways and functional information of genic sequences originated piRNA-associated genes.

Gene symbol	Pathways	Functional domains	Molecular function gene ontologies	Functions related to germ cells, testis, and ovary	References for gene functions
CHIR-B5	-	Immunoglobulin domain 2; Immunoglobulin domain 3	-	-	
CHIR-AB1	-	Immunoglobulin domain 2	-	-	
PLLP	-	Myelin and lymphocyte protein (MAL) and related proteins for vesicle trafficking and membrane link (MARVEL) domain	-	-	
MYO1A	-	Myosin head; Isoleucine and glutamine calmodulin-binding motifs; Myosin tail	Actin binding; ATP binding; ATPase activity; Calmodulin binding; Microfilament motor activity	Testes handedness in *Drosophila*.	[1]
SLC6A2	-	Sodium-neurotransmitter symporter family (SNF) domain	Sodium-neurotransmitter symporter	Feedback regulation of luteinizing hormone in mammals.	[2]
17.5	-	Lectin C-type domain	-	-	
RAP2B	-	Ras family domain	GTP binding; GTPase activity	Rearrangement of the pigment granules in *Xenopus* oocytes.	[3]
LRP8	-	Low-density lipoprotein receptor domain class A and B domains; Coagulation factor Xa inhibitory site; Calcium-binding EGF domain	Calcium ion binding; Protein binding	Maintenance of mitochondrial sheath in mice sperms.<br><br>Regulates follicular growth, and	[4-6]

				cholesterol delivery to theca and granulosa cells in bovine ovary.	
VDR	Endocrine and other factor-regulated calcium reabsorption; Mineral absorption	Zinc finger, C4 type domain; Ligand-binding domain of nuclear hormone receptor	Calcitriol receptor activity; sequence-specific DNA binding; Steroid hormone receptor activity; Zinc ion binding	Vitamin D3 metabolism, and Ca^{2+} homeostasis in several reproductive cells of mammals, birds, and Zebrafish.	[7-10]
MYH1E	Tight junction	Myosin N-terminal SH3-like domain; Myosin head; Myosin tail	ATP binding; Motor activity	Induction of cortical granule exocytosis in *Xenopus* oocytes.	[11]
ZNF302	-	Kruppel-associated box domain; Zinc-finger double domains	Metal ion binding; Nucleic acid binding	Predominantly have a role on spermatogenesis in human. Induces apoptosis in human ovarian cancer cells.	[12, 13]
RGN	Carbon metabolism; Degradation of aromatic compounds; Pentose phosphate pathway; Ascorbate and aldarate metabolism	SMP-30/gluconolaconase/LRE-like region	Calcium ion binding; Enzyme regulator activity; Gluconolactonase activity; Zinc ion binding	Ca^{2+} homeostasis and control of gametogenesis in mammals and birds.	[14, 15]
FOXD2	-	Fork head domain	-	-	
RASSF2	-	Ras associateion (RalGDS/AF-6) domain	-	Tumor suppressor in human ovarian cancer.	[16]
VAMP7	SNARE interactions in vesicular transport	Regulated-SNARE-like domain; Synaptobrevin domain	-	Colocalize with a tumor protein D_{52} in Ca^{2+}-regulated membrane trafficking in Chinese hamster	[17]

				ovarian cells.	
NLGN1	Cell adhesion molecules	Carboxylesterase family domain	-	-	
APOA1	Peroxisome proliferator-activated receptor signaling pathway	Apolipoprotein A1/A4/E domain	Apolipoprotein A1 receptor binding; beta-amyloid binding; Cholesterol transporter activity; High-density lipoprotein particle/receptor binding; Phosphatidylcholine-sterol O-acyltransferase activator; Phospholipid binding	Cholesterol uptake and efflux in steroidogenic cells and germ cells in mammals.<br><br>Marker to detect metabolic syndrome in women with polycystic ovarian syndrome.<br><br>Marker to trace DNA methylation pattern from the germ cells through the early embryo to adult tissues.	[18-21]
ADK	Purine metabolism	Phosphofructokinase isozyme B family carbohydrate kinase	Adenosine kinase activity; Phosphotransferase activity, alcohol group as acceptor	-	

References

1. Hozumi S, Maeda R, Taniguchi K, Kanai M, Shirakabe S, Sasamura T, Speder P, Noselli S, Aigaki T, Murakami R, Matsuno K: **An unconventional myosin in Drosophila reverses the default handedness in visceral organs**. *Nature* 2006, **440**:798-802.
2. Yang SP, Pau KY, Spies HG: **Gonadectomy alters tyrosine hydroxylase and norepinephrine transporter mRNA levels in the locus coeruleus in rabbits**. *J Neuroendocrinol* 1997, **9**:763-768.
3. Campa MJ, Farrell FX, Lapetina EG, Chang KJ: **Microinjection of Rap2B protein or RNA induces rearrangement of pigment granules in Xenopus oocytes**. *Biochem J* 1993, **292**:231-236.

4. Suzuki-Toyota F, Ito C, Toyama Y, Maekawa M, Yao R, Noda T, Iida H, Toshimori K: **Factors maintaining normal sperm tail structure during epididymal maturation studied in Gopc-/- mice**. *Biol Reprod* 2007, **77**:71-82.
5. Fayad T, Lefebvre R, Nimpf J, Silversides DW, Lussier JG: **Low-density lipoprotein receptor-related protein 8 (LRP8) is upregulated in granulosa cells of bovine dominant follicle: molecular characterization and spatio-temporal expression studies**. *Biol Reprod* 2007, **76**:466-475.
6. Argov N, Sklan D: **Expression of mRNA of lipoprotein receptor related protein 8, low density lipoprotein receptor, and very low density lipoprotein receptor in bovine ovarian cells during follicular development and corpus luteum formation and regression**. *Mol Reprod Dev* 2004, **68**:169-175.
7. Dornas RA, Oliveira AG, Kalapothakis E, Hess RA, Mahecha GA, Oliveira CA: **Distribution of vitamin D3 receptor in the epididymal region of roosters (*Gallus domesticus*) is cell and segment specific**. *Gen Comp Endocrinol* 2007, **150**:414-418.
8. Craig TA, Sommer S, Sussman CR, Grande JP, Kumar R: **Expression and regulation of the vitamin D receptor in the zebrafish, Danio rerio**. *J Bone Miner Res* 2008, **23**:1486-1496.
9. Lerchbaum E, Obermayer-Pietsch B: **Vitamin D and fertility: a systematic review**. *Eur J Endocrinol* 2012, **166**:765-778.
10. Smolikova K, Mlynarcikova A, Scsukova S: **Effect of 1alpha,25-dihydroxyvitamin D3 on progesterone secretion by porcine ovarian granulosa cells**. *Endocr Regul* 2013, **47**:123-131.
11. Schietroma C, Yu HY, Wagner MC, Umbach JA, Bement WM, Gundersen CB: **A role for myosin 1e in cortical granule exocytosis in Xenopus oocytes**. *J Biol Chem* 2007, **282**:29504-29513.
12. Goldwurm S, Menzies ML, Banyer JL, Powell LW, Jazwinska EC: **Identification of a novel Krueppel-related zinc finger gene (ZNF184) mapping to 6p21.3**. *Genomics* 1997, **40**:486-489.
13. Huang C, Yang S, Ge R, Sun H, Shen F, Wang Y: **ZNF23 induces apoptosis in human ovarian cancer cells**. *Cancer Lett* 2008, **266**:135-143.
14. Bruggeman V, Van den Bergh G, Clerens S, Dumez L, Onagbesan O, Arckens L, Decuypere E: **Effect of a single in ovo injection of 2,3,7,8-tetrachlorodibenzo-p-dioxin on protein expression in liver and ovary of the one-day-old chick analyzed by fluorescent two-dimensional difference gel electrophoresis and mass spectrometry**. *Proteomics* 2006, **6**:2576-2585.
15. Laurentino SS, Correia S, Cavaco JE, Oliveira PF, de Sousa M, Barros A, Socorro S: **Regucalcin, a calcium-binding protein with a role in male reproduction?** *Mol Hum Reprod* 2012, **18**:161-170.
16. Lambros MB, Fiegler H, Jones A, Gorman P, Roylance RR, Carter NP, Tomlinson IP: **Analysis of ovarian cancer cell lines using array-based comparative genomic hybridization**. *J Pathol* 2005, **205**:29-40.
17. Thomas DD, Martin CL, Weng N, Byrne JA, Groblewski GE: **Tumor protein D52 expression and Ca2+-dependent phosphorylation modulates lysosomal membrane protein trafficking to the plasma membrane**. *Am J Physiol Cell Physiol* 2010, **298**:C725-739.

18. Ariel M, Cedar H, McCarrey J: **Developmental changes in methylation of spermatogenesis-specific genes include reprogramming in the epididymis**. *Nat Genet* 1994, **7**:59-63.
19. Plump AS, Erickson SK, Weng W, Partin JS, Breslow JL, Williams DL: **Apolipoprotein A-I is required for cholesteryl ester accumulation in steroidogenic cells and for normal adrenal steroid production**. *J Clin Invest* 1996, **97**:2660-2671.
20. Yin Q, Chen X, Li L, Zhou R, Huang J, Yang D: **Apolipoprotein B/apolipoprotein A1 ratio is a good predictive marker of metabolic syndrome and pre-metabolic syndrome in Chinese adolescent women with polycystic ovary syndrome**. *J Obstet Gynaecol Res* 2013, **39**:203-209.
21. Shemer R, Kafri T, O'Connell A, Eisenberg S, Breslow JL, Razin A: **Methylation changes in the apolipoprotein AI gene during embryonic development of the mouse**. *Proc Natl Acad Sci U S A* 1991, **88**:11300-11304.
