# Supplementary material for: Small non-coding RNA profiling and the role of piRNA pathway genes in the protection of chicken primordial germ cells
Source: BMC Genomics. 2014 Sep 4;15(1):757. doi: 10.1186/1471-2164-15-757 (PMC4286946; doi:10.1186/1471-2164-15-757)
Supplement: Supplementary file 8 — Additional file 8: Table S5: Survival rate of PGCs purified by MACS. (PDF 39 KB) [file 12864_2014_6778_MOESM8_ESM.pdf]

Table S5. The survival rate of PGCs purified by MACS.

Trials	Number of embryos used	Total number of cells isolated from embryonic gonads	Number of cells after MACS[1]	Viability (%)[2]
T1	554	6.3×10^7	593000	97
T2	577	7.3×10^7	689000	98
T3	586	8.7×10^7	813000	96
T4	326	3.1×10^7	299000	97

[1]Number of SSEA1-positive cells after MACS

[2]Percentage of live cells by trypan blue staining
