# Supplementary material for: Small non-coding RNA profiling and the role of piRNA pathway genes in the protection of chicken primordial germ cells
Source: BMC Genomics. 2014 Sep 4;15(1):757. doi: 10.1186/1471-2164-15-757 (PMC4286946; doi:10.1186/1471-2164-15-757)
Supplement: Supplementary file 9 — Additional file 9: Table S6: qPCR primers used for the amplification of piRNAs. (PDF 41 KB) [file 12864_2014_6778_MOESM9_ESM.pdf]

Table S6. qPCR primers used for the amplification of piRNAs.

Seq. ID	Primer Sequences (5’-3’)	Seq. ID	*Primer Sequences (5’-3’)
ISG_3439104	F: TATTTCCTAACGTCCAGCCTG	ISG_1952422	F: AGAACACACTTGGCCTTCC
ISG_1920655	F: ACTGAACACAGCACTCGAGGT	ISG_2943457	F: AGAGCTGCCCCTGAGCCT
ISG_1259042	F: GAAGATGTGCACCTGACGC	ISG_2785619	F: GCATGTTGCTCTCTGTCAGC
ISG_2022559	F: GTTTACTGACTGAGCTACTTTTCCC	ISG_2633063	F: GGTACTGAGACATCTTGGAGACAA
ISG_1354003	F: TCCAAGGACCAGTAGCGCT	ISG_2087909	F: CTCAAAGGATTCCGCATCG
ISG_3065006	F: CCAAATGGTAGCAGAGGCC	ISG_3356981	F: GCTGAGAACTAAGGATGCCTCC
ISG_3294350	F: TGTGACCCTGGAATTCCACTAC	ISG_2645108	F: GTGAGAAGGGCTTTGTGCAG
ISG_2675670	F: GTGTGGGACGAGAAGGAGAAC	ISG_3080707	F: GTGTGTGAGTGCGGCGG
ISG_3280151	F: AAGGACCTCTGAGAATTGCTTTCT	ISG_1621199	F: AAGAGAAGCTGAACACAGGGTG
snoRNA	F: GGGATGTAAAAAAATACTTGCTATC		

*F: Forward primers. Universal reverse primer was purchased from Agilent Technologies.
