# Supplementary material for: Small non-coding RNA profiling and the role of piRNA pathway genes in the protection of chicken primordial germ cells
Source: BMC Genomics. 2014 Sep 4;15(1):757. doi: 10.1186/1471-2164-15-757 (PMC4286946; doi:10.1186/1471-2164-15-757)
Supplement: Supplementary file 10 — Additional file 10: Table S7: qPCR primers used for the amplification of genes. (PDF 78 KB) [file 12864_2014_6778_MOESM10_ESM.pdf]

Table S7. qPCR primers used for the amplification of genes.

Gene Symbol	Description	GenBank Accession no.	Forward Primer (5'-3')	Reverse Primer (5'-3')
CR1	Retrotransposon CR1	U88211	AAACTCCATGGGCAAAGATGGTG	GGCTCTCGACCTGTTCCTGACTG
CHIR-B5	Immunoglobulin-like receptor CHIR-B5	NM_001146136	GAGCATGTGGAGATGGGGAG	AAAGAGGGTGAAGGTGGCAG
CHIR-AB1	Immunoglobulin-like receptor CHIR-AB1	NM_001146141	GACACGGAGAGATGAGAGCG	TAGATGACAGGGGACTGGGG
PLLP	Plasmolipin	NM_001030561	GCAGCTCCCCATGAAGTTCT	CAGCGGCTCTCCTGTTGTAA
MYO1A	Myosin IA	NM_205163	TGCTGATGATGCGTATCGCT	CACTTTGTCCACCTCCTCCC
SLC6A2	Solute carrier family 6 (neurotransmitter transporter, noradrenalin), member 2	NM_204716	AGTACACCAGCAGCAACCTC	CAGAGGTAGGGGAACCTCCA
17.5	Lectin-like protein, type II transmembrane protein (17.5)	NM_205429	AGCTGAGACAGAACAGGTGC	GGAAACCGATTGAGGCGTTG
RAP2B	RAP2B, member of RAS oncogene family	NM_001030702	ACGGACAGACGGACGGACAG	CGGCACCCAAGAAGACCAAG
LRP8	Low density lipoprotein receptor-related protein 8, apolipoprotein e receptor	NM_205186	GGCTGGGTCCTGACATGAAA	TTCCGATACAGCTCTGGGGA
VDR	Vitamin D (1,25- dihydroxyvitamin D3) receptor	NM_205098	AGTCAGCGATGTCACCCAAG	GATCTGGGGACAGGATGCAG
MYH1E	Myosin, heavy chain 1E, skeletal muscle	NM_001013397	GCCCTGGATGAAGCTGTTCT	CTCCTCCAGCTCCTTCCTCT
ZNF302	Zinc finger protein 302;	NM_001030695	GAGCAGCTGTGTCTCAGAGG	GCTGCTTTTGAGCTGTGCTT
RGN	Regucalcin	NM_204729	CTTCCTGCTGCTTTGGAGGA	CACACCAAGCCCAGTGATCT

FOXD2	Forkhead box D2	NM_204952	GACATGTTCGACAACGGCAG	GCCGTAGTTGTAGCCGTAGG
RASSF2	Ras association (RalGDS/AF-6) domain family member 2	NM_001030884	CTGTGAGCAGGTCTCCAAGG	CTTCTTGACTTCGCGGTCCT
VAMP7	Vesicle-associated membrane protein 7	NM_001031121	AAGAGCACAGACAGCCCTTC	CTACCTGGTCAGTGCCCTTG
CIWI	Chicken PIWI (P-element-induced wimpy testis)-like protein 1	NM_001098852	CCGAAATGGAGAAGATGTGAGGA	TGTGATTAGGGATGCTGACTGGG
CILI	Chicken PIWI (P-element-induced wimpy testis)-like protein 2	JN248386	GCTCCGTCATCGGCTTCGT	TTCTTGGGCAGGCAGTGGTT
GAPDH	Glyceraldehyde-3-phosphate dehydrogenase	NM_204305	CCTCTCTGGCAAAGTCCAAG	CATCTGCCCATTTGATGTTG
